# A genome-wide association meta-analysis on apolipoprotein A-IV concentrations

**DOI:** 10.1093/hmg/ddw211

**Published:** 2016-07-12

**Authors:** Claudia Lamina, Salome Friedel, Stefan Coassin, Rico Rueedi, Noha A. Yousri, Ilkka Seppälä, Christian Gieger, Sebastian Schönherr, Lukas Forer, Gertraud Erhart, Barbara Kollerits, Pedro Marques-Vidal, Janina Ried, Gerard Waeber, Sven Bergmann, Doreen Dähnhardt, Andrea Stöckl, Stefan Kiechl, Olli T. Raitakari, Mika Kähönen, Johann Willeit, Ludmilla Kedenko, Bernhard Paulweber, Annette Peters, Thomas Meitinger, Konstantin Strauch, KORA Study Group, Terho Lehtimäki, Steven C. Hunt, Peter Vollenweider, Florian Kronenberg

**Affiliations:** 1 Division of Genetic Epidemiology, Department of Medical Genetics, Molecular and Clinical Pharmacology, Medical University of Innsbruck, Innsbruck, Austria; 2 Department of Neurology, Medical University of Innsbruck, Innsbruck, Austria; 3 Department of Computational Biology, University of Lausanne, Lausanne, Switzerland; 4 Swiss Institute of Bioinformatics, Lausanne, Switzerland; 5 Department of Physiology and Biophysics, Weill Cornell Medical College − Qatar, Doha, Qatar; 6 Department of Computer and Systems Engineering, Alexandria University, Alexandria, Egypt; 7 Department of Clinical Chemistry, Fimlab Laboratories and University of Tampere School of Medicine, Tampere, Finland; 8 Institute of Genetic Epidemiology, Helmholtz Zentrum München—German Research Center for Environmental Health; 9 Institute of Epidemiology II, Helmholtz Zentrum München—German Research Center for Environmental Health; 10 Research Unit of Molecular Epidemiology, Helmholtz Zentrum München—German Research Center for Environmental Health, Neuherberg, Germany; 11 Department of Medicine, Internal Medicine, Centre Hospitalier Universitaire Vaudois (CHUV), Lausanne, Switzerland; 12 Department of Clinical Physiology, Turku University Hospital, Turku, Finland; 13 Research Centre of Applied and Preventive Cardiovascular Medicine, University of Turku, Turku, Finland; 14 Department of Clinical Physiology, Tampere University Hospital and University of Tampere, Tampere, Finland; 15 First Department of Internal Medicine, Paracelsus Private Medical University, Salzburg, Austria; 16 DZHK (German Centre for Cardiovascular Research), partner site Munich Heart Alliance, Munich, Germany; 17 German Center for Diabetes Research (DZD e.V.), Neuherberg, Germany; 18 Institute of Human Genetics, Technische Universität München, München, Germany; 19 Institute of Human Genetics, Helmholtz Zentrum München, Neuherberg, Germany; 20 Munich Cluster for Systems Neurology (SyNergy); 21 Institute of Medical Informatics, Biometry and Epidemiology, Chair of Genetic Epidemiology, Ludwig-Maximilians-Universität, Munich, Germany; 22 Cardiovascular Genetics Division, University of Utah School of Medicine, Salt Lake City, UT, USA; 23 Department of Genetic Medicine, Weill Cornell Medicine, Doha, Qatar

## Abstract

Apolipoprotein A-IV (apoA-IV) is a major component of HDL and chylomicron particles and is involved in reverse cholesterol transport. It is an early marker of impaired renal function. We aimed to identify genetic loci associated with apoA-IV concentrations and to investigate relationships with known susceptibility loci for kidney function and lipids. A genome-wide association meta-analysis on apoA-IV concentrations was conducted in five population-based cohorts (*n =* 13,813) followed by two additional replication studies (*n =* 2,267) including approximately 10 M SNPs. Three independent SNPs from two genomic regions were significantly associated with apoA-IV concentrations: rs1729407 near *APOA4* (*P =* 6.77 × 10 ^−^ ^44^), rs5104 in *APOA4* (*P =* 1.79 × 10^−^^24^) and rs4241819 in *KLKB1* (*P =* 5.6 × 10^−^^14^). Additionally, a look-up of the replicated SNPs in downloadable GWAS meta-analysis results was performed on kidney function (defined by eGFR), HDL-cholesterol and triglycerides. From these three SNPs mentioned above, only rs1729407 showed an association with HDL-cholesterol (*P =* 7.1 × 10 ^−^ ^07^). Moreover, weighted SNP-scores were built involving known susceptibility loci for the aforementioned traits (53, 70 and 38 SNPs, respectively) and were associated with apoA-IV concentrations. This analysis revealed a significant and an inverse association for kidney function with apoA-IV concentrations (*P =* 5.5 × 10^−^^05^). Furthermore, an increase of triglyceride-increasing alleles was found to decrease apoA-IV concentrations (*P =* 0.0078). In summary, we identified two independent SNPs located in or next the *APOA4* gene and one SNP in *KLKB1*. The association of *KLKB1* with apoA-IV suggests an involvement of apoA-IV in renal metabolism and/or an interaction within HDL particles. Analyses of SNP-scores indicate potential causal effects of kidney function and by lesser extent triglycerides on apoA-IV concentrations.

## Introduction

Apolipoprotein A-IV (apoA-IV) is an antioxidative glycoprotein that is synthesized primarily in the intestine and to a lesser extent in the liver (1,2). It is secreted into the lymph as a structural protein of chylomicrons, very-low-density lipoproteins, high-density lipoproteins and participates in reverse cholesterol transport (3,4). Consequently, it plays an important role in relieving peripheral cells of an overload of cholesterol (5,6). It has an effect on fat resorption and has been discussed to be a satiety factor and related to diet-induced obesity at least in animal models (2). ApoA-IV shows anti-atherogenic properties (7,8) and low concentrations were found to be associated with cardiovascular outcomes (9–12). Moreover, it acts as an early marker of impaired renal function and is a predictor of a progression of chronic kidney disease (13–16).

The knowledge on the genetic regulation of apoA-IV is limited. Heritability estimates derived from a family-based study in 119 nuclear families varied between 0% and 67%, depending on the underlying model (17). ApoA-IV is expressed by the *APOA4* gene on chromosome 11. This gene is in close proximity and linkage with *APOA5*, *APOC3* and *APOA1*. This gene region is often referred as the *APOA5-A4-C3-A1* gene cluster. There have been numerous candidate gene studies, which primarily evaluated the non-synonymous variants rs675 (T347S) and rs5110 (Q360H) with e.g. the ability of apoA-IV to bind lipids and to promote cholesterol efflux from cells (18). Association results of these variants with plasma apoA-IV levels (19,20) as well proposed associations with triglycerides were contradictory (21–23). Variants in the *APOA5-A4-C3-A1* gene cluster have also been found to be associated on a genome-wide scale with lipid phenotypes, primarily with triglyceride and HDL cholesterol (HDL-C) concentrations (24). Up to now, there have been no genome-wide studies (GWAS) investigating apoA-IV concentrations.

The aim of the present study was to identify gene loci that are associated with apoA-IV concentrations based on a hypothesis-free approach. We conducted a genome-wide association meta-analysis using data from five population-based studies followed by a replication step in two additional studies. We also performed gene-based and pathway analyses to shed new light on the functional role of the identified genes and/or apoA-IV. Since the information on the heritability of apoA-IV is limited, we conducted a polygenic analysis to calculate the heritability of apoA-IV concentrations as well as the proportion of phenotypic variance explained by the single nucleotide polymorphisms (SNPs). ApoA-IV is known to be associated with kidney function and lipid phenotypes. Therefore, we also performed look-ups in and from the respective GWAS to elucidate possible causal relationships.

## Results

### Description of cohorts and quality control

**Figure 1.**
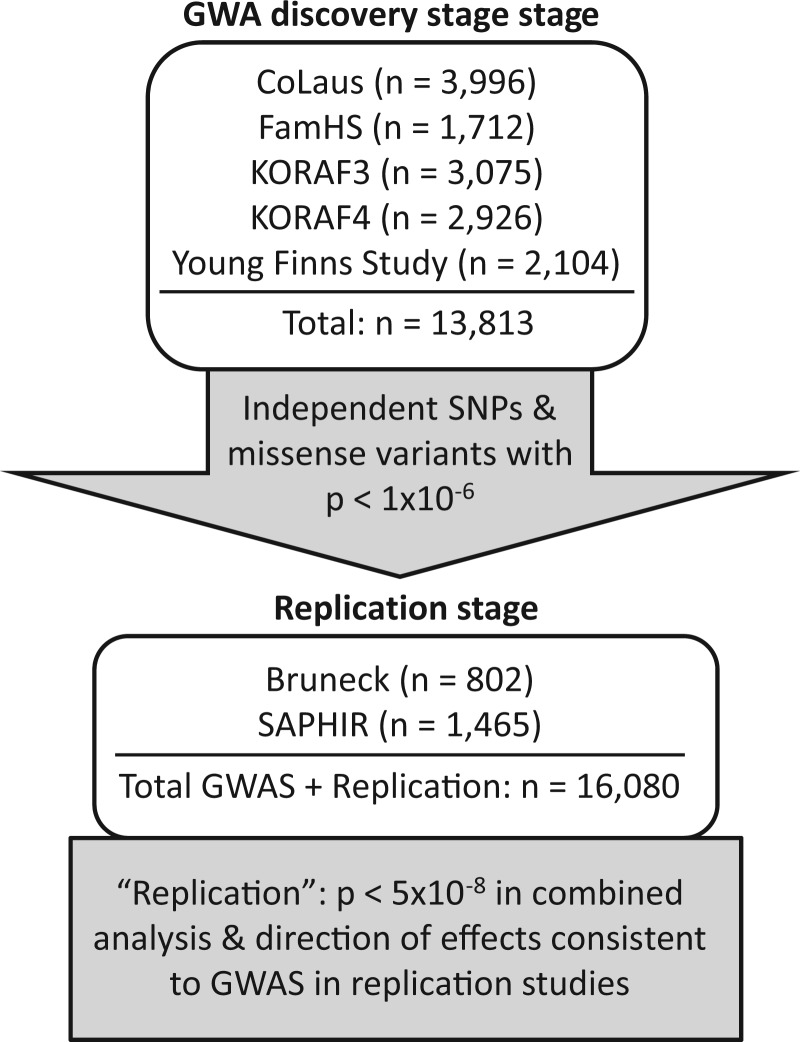
Overview of contributing cohorts in the discovery and replication stage.

Five studies contributed to the discovery stage (*n =* 13,813) and 2 additional studies to the replication phase (*n =* 2,267) (Figure 1), altogether including data from 16,080 participants. Due to the skewed distribution of apoA-IV concentrations, log-transformation of values was performed in all studies, resulting in nearly normal distributions (Supplementary Material, Figure 1). Descriptive characteristics of all studies can be found in Supplementary Material, Table 1. Meta-Analysis and quality control was performed as described in Supplementary Material, Figure 2. The P-Z-plot did not reveal any deviations of the reported *P*-values and *P*-values calculated by the beta coefficient and standard error. Genomic inflation factors λ within studies ranged from 1.011 to 1.038 (Supplementary Material, Table 2).

### GWA discovery stage

**Figure 2.**
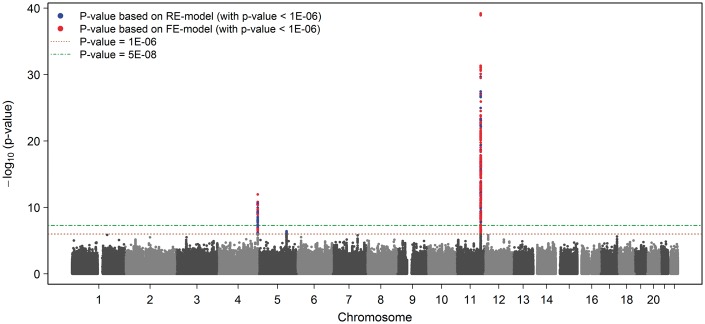
Manhattan-plot for the meta-analysis on log-transformed apoA-IV values. Results are based on five discovery cohorts including 13813 individuals.

**Figure 3.**
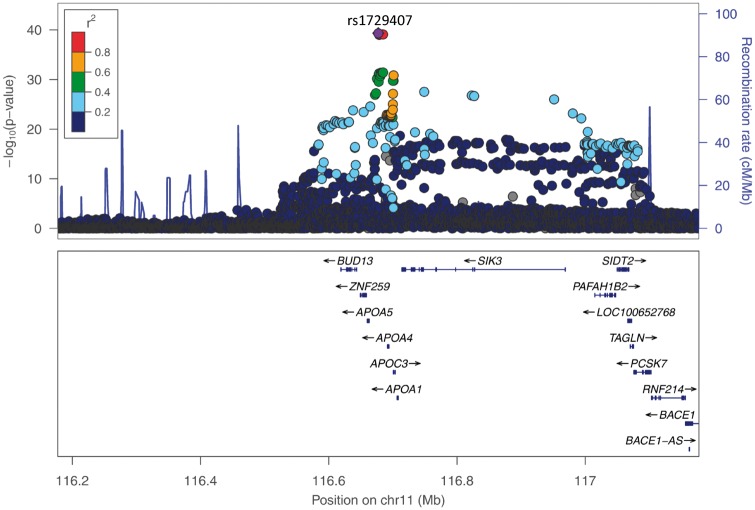
Regional plot showing the genomic region defined by the *APOA4*-lead SNP rs1729407 +/- 500 kB (LD refers to rs1729407, based on 1000G EUR); *P*-values are derived from the meta-analysis on the five discovery cohorts on log-transformed apoA-IV concentrations.

**Figure 4.**
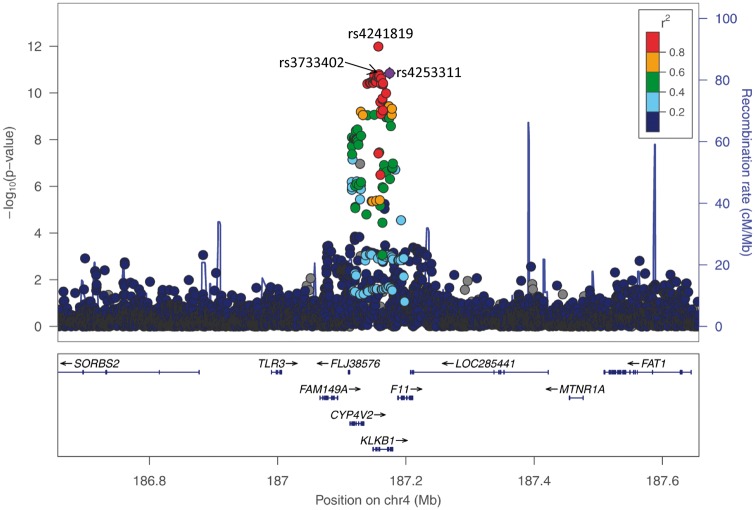
Regional plot showing the genomic region defined by the *KLKB1*-lead SNP rs4241819 +/- 500 kB (LD is based on 1000G EUR and refers to rs4253311 which was used as a proxy for the lead SNP in the replication studies); additionally, the missense variant rs3733402 is marked. *P*-values are derived from the meta-analysis on the five discovery cohorts on log-transformed apoA-IV concentrations.

The GWA meta-analysis (stage 1) resulted in two genome-wide significant gene-regions (Manhattan plot shown in Figure 2, QQ-plot shown in Supplementary Material, Figure 3). In a broad region surrounding the *APOA4* gene, 423 SNPs reached genome-wide significance with the lowest *P*-value for SNP rs1729407 (*P =* 6.00×10^−^^40^, Figure 3). Additionally, 64 genome-wide significant SNPs in the gene-region around the *KLKB1* gene on chromosome 4 were identified (lowest *P*-value for SNP rs4241819: 1.08×10^−^^12^, Figure 4). Furthermore, one locus on chromosome 5 (*SOWAHA*) reached our predefined level of significance sufficient for replication using the RE model by Han & Eskin (lowest *P*-value for SNP rs59698941: 3.76×10^−^^07^, Supplementary Figure 4).

### SNP selection

Conditional analyses were performed for *APOA4* (chr11: 116177370-117177370, Figure 3), *KLKB1* (chr4: 186657140-187657140, Figure 4) and *SOWAHA* (chr5: 131654912-132654912, Supplementary Material, Figure 4). For *APOA4*, two SNPs were independently associated with apoA-IV concentrations: rs1729407 (*P*-value single SNP analysis: 6.00 × 10^−^^40^; *P*-value conditional analysis: 2.66 × 10^−^^25^) and rs5104 (*P*-value single SNP analysis: 1.24 × 10^−^^22^; *P*-value conditional analysis: 4.01 × 10^−^^08^, Supplementary Material, Figure 5) . After apoA-IV concentrations were adjusted for these two SNPs, no further SNPs remained in the model with an adjusted *P*-value less than 1x10^−^^6^ (Supplementary Material, Figure 6). Besides the two SNPs rs1729407 and rs5104 the following missense variants were selected for replication: rs5110 (*P =* 9.26 × 10^−^ ^07^) and rs675 (*P =* 0.0021). The latter was selected due to its wide use in the literature. For *KLKB1* and *SOWAHA*, no additional SNP was added by applying the conditional analysis. One missense variant was selected within the *SOWAHA* gene region (rs2292030, *P =* 9.24 × 10^−^^07^ using the RE model, within *SHROOM1*). The lead SNP in *KLKB1* (rs4241819) and the *KLKB1* missense variant rs3733402 that were selected for replication were not accessible to iPLEX genotyping. Therefore, a proxy SNP (rs4253311) in high linkage disequilibrium (LD) with both the lead SNP and the missense variant was chosen for replication (*P =* 1.43 × 10^−^^11^, *r*^2 ^=^ ^0.932 with rs4241819, *r*^2 ^=^ ^0.994 with rs3733402, based on 1000 Genomes phase 3 v5; see also Figure 4 for graphical display of LD between the SNPs). Characteristics of all selected SNPs can be found in Supplementary Material, Table 3.

### Replication stage and combined analysis

Altogether, 7 SNPs were genotyped in the two replication studies. The single study results for these SNPs are given in Supplementary Material, Table 4. Of these, 3 SNPs reached a false-discovery rate less than 0.05 on the replication stage and a genome-wide significance level after inclusion of all 7 studies (discovery stage + replication stage, Table 1): rs1729407 near *APOA4* (*P =* 6.77 × 10^−^^44^), rs5104 in *APOA4* (*P =* 1.79 × 10^−^^24^) and rs4241819 (using rs4253311 as proxy in the replication studies) in *KLKB1* (*P =* 5.63×10^−^^14^). For these 3 SNPs, effect directions were identical in all studies. For each copy of the minor allele of rs1729407, apoA-IV concentrations decrease by 0.2645 mg/dl. Each minor allele of rs5104 also decreases apoA-IV concentrations by 0.2526 mg/dl. In a joint analysis, both SNPs remain significant (*P =* 2.66 × 10^−^^25^ for rs1729407, *P =* 4.01 × 10^−^^08^ for rs5104) with slightly smaller effect estimates (β = 0.2041 for rs1729407 and β = 0.1455 for rs5104). The minor allele of SNP rs4241819/rs4253311 in *KLKB1* increases apoA-IV concentrations by 0.1469 mg/dl.

**Table 1.** Meta-analysis results of selected SNPs for replication (*P*-value < 1E-06 in the GWAs stage); the beta estimate and effect direction refer to the minor allele

	** GWAS stage **				** Replication stage **					** GWAS ± Replication **		
SNP	β (se)*	**Effect Direction**^§^	P	**I**^2^	β (se)*	**Effect Direction**^§^			**p/FDR**^$^	β (se)*	p	**I**^2^
***APOA4* gene region**												
rs1729407	−0.2459 (0.0289)	− − − − −	6.00E-40	0	−0.4895 (0.1003)	− −	3.73E-06/2.59E-05			−0.2645 (0.0277)	6.77E-44	25.34
rs5104	−0.2399 (0.0367)	− − − − −	1.24E-22	15.63	−0.4533 (0.1460)	− −	0.0013/0.0046			−0.2526 (0.0356)	1.79E-24	11.05
rs5110	0.2301 (0.0774)	++ +++	9.26E-07	0	0.0520 (0.1956)	− +	0.9124/0.9124			0.2060 (0.0720)	1.44E-05	1.37
***APOA4* gene region, selected from literature**												
rs675	−0.1041 (0.0380)	− − − − −	0.0021	0	−0.1462 (0.1183)	− −		0.2931/0.5129		−0.1081 (0.0362)	0.0013	0
**Other gene regions**												
*KLKB1*: rs4241819/rs4253311	0.1395 (0.0280)	++ +++	1.08E-12	45.89	0.2410 (0.1006)	++		0.0093/0.0217		0.1469 (0.0270)	5.63E-14	36.03
*SOWAHA:* rs59698941	−0.3542 (0.1420)^#^	− − − − −	3.76E-07^&^	68.83	−0.0162 (0.1638)^#^	+ −		0.6637^&/^0.7743		−0.2628 (0.1111) ^#^	1.75E-06^&^	64.86
*SHROOM1:* rs2292030	−0.3502 (0.1407)^#^	− − − − −	9.24E-07^&^	66.33	−0.0222 (0.1491)^#^	+ −		0.6584^&/^0.7743		−0.2629 (0.1090)^#^	4.28E-06^&^	59.86

* All effect estimates (β and se) are based on the original scale of apoA-IV, either fixed effect or random effect. Where labeled, all effect estimates refer to the minor allele derived from 1000G, phase 3v5 (see Supplementary Material, Table 3).

^§^Order of included GWA studies: CoLaus, FamHS, KORA F3, KORA F4, YFS. +: positive effect from minor allele on log(apoA-IV) in that specific study, −: inverse effect from minor allele on log(apoA-IV) in that specific study;?: SNP not available in that study; Order of included studies at replication stages: Bruneck, SAPHIR.

^&^*P*-values are derived from the method proposed by Han and Eskin.

^#^Random effects β and se.

^$^False-discovery rate by Benjamini and Hochberg (50).

### Effects in men and women

GWAS stratified for men and women did not reveal any additional genome-wide significant SNPs outside the wider *APOA4* gene region (Supplementary Material, Figures 7 and 8). There was also no genome-wide significant SNP-gender interaction effect (Supplementary Material, Figure 9).

### APOA-IV variance explained

All SNPs combined within the broad *APOA4* region (lead SNP +/- 500 kB) explained ∼3.30% (95% CI: [1.60%; 5.00%]) of the phenotypic variance assuming an additive model, based on both KORA studies. The top SNP rs1729407 alone explained 1.38% in KORAF3/F4 and 1.39% in the SAPHIR study, respectively, and rs5104 alone 1% in KORAF3/F4 and 0.57% in SAPHIR. The *KLKB1* region (lead SNP +/- 500 kB) accounted for 0.67% (95% CI: [0.00%; 1.54%]) in KORAF3/F4. SNP rs4241819/rs4253311 alone explained 0.44% in KORAF3/F4 and 0.19% in the SAPHIR study, respectively. All three SNPs together (two in *APOA4*, one in *KLKB1*) in one model explained 2.2% in the combined KORAF3/F4 dataset. The genome-wide SNP-based explained variance including the entire dataset of available SNPs (genomic heritability) was estimated to be 36.07% in both KORA studies (95% CI: [18.48%; 53.66%]). The narrow-sense heritability h^2^ of apoA-IV, derived from the polygenic model in the family-based FamHS study, was estimated to be 27.45%.

### Gene-based and pathway analyses

The gene-based association scan resulted in 18 significant genes, all of which are located either in the broad *APOA4* or *KLKB1* gene regions (Table 2). The pathway analysis revealed 15 gene sets to be significantly enriched with susceptibility genes, including expected lipid transport and lipoprotein metabolism pathways as well as some additional liver-related pathways (Supplementary Material, Table 5).

**Table 2.** Results of gene-based analysis

Gene	Nominal *P*-value	Bonferroni-corrected *P*-value	Chromosome	Position	Group
*ZPR1*	7.93E-39	1.9E-34	11	116649275	protein-coding gene
*APOA5*	2.14E-38	5.38E-34	11	116660085	protein-coding gene
*APOA4*	4.06E-38	1.02E-33	11	116691417	protein-coding gene
*APOC3*	7.2E-38	1.81E-33	11	116700623	protein-coding gene
*APOA1*	1.23E-29	3.09E-25	11	116706468	protein-coding gene
*APOA1-AS*	1.53E-29	3.84E-25	11	116706832	non-coding RNA
*SIK3*	2.78E-29	6.99E-25	11	116714117	protein-coding gene
*BUD13*	5.57E-23	1.40E-18	11	116618885	protein-coding gene
*PAFAH1B2*	1.32E-18	3.32E-14	11	117014999	protein-coding gene
*SIDT2*	1.08E-16	2.71E-12	11	117049938	protein-coding gene
*TAGLN*	1.69E-16	4.25E-12	11	117070039	protein-coding gene
*LOC100652768*	1.85E-16	4.65E-12	11	117066328	unknown
*PCSK7*	3.59E-16	9.02E-12	11	117075786	protein-coding gene
*KLKB1*	5.86E-11	1.47E-06	4	187148671	protein-coding gene
*RNF214*	3.24E-10	8.14E-06	11	117103451	protein-coding gene
*F11*	9.03E-10	2.27E-05	4	187187117	protein-coding gene
*CYP4V2*	1.21E-09	3.04E-05	4	187112673	protein-coding gene
*FLJ38576*	3.35E-08	8.42E-04	4	187110185	unknown

### Look-up in other GWA meta-analysis consortia

We looked up the two replicated SNPs in the *APOA4* gene region (rs1729407, rs5104), the replicated SNP in *KLKB1* (rs4241819), its proxy used in the replication step (rs4253311) and the correlated missense mutation (rs3733402) in the GWA meta-analysis results on kidney function, HDL-C and triglycerides. Two SNPs (rs1729407, rs4253311) were available in all GWAS consortia. Only one significant result was found: the apoA-IV lead SNP rs1729407 was associated with HDL-C with a *P*-value of 7.1×10 ^−^ ^07^ (Table 3).

**Table 3.** Look-up of lead apoA-IV SNPs in other consortia, providing *P*-values

	*P***-value of association with…**		
SNPs	TG	HDL-C	eGFR
rs1729407 (*APOA4*)	0.96	7.1E-07	0.73
rs4253311 (*KLKB1*)	0.07	0.54	0.17

**Table 4.** Association of weighted SNP-scores including susceptibility SNPs for kidney function, HDL cholesterol and triglycerides (TG) with log-transformed apoA-IV levels (age-and sex-adjusted) in the combined KORAF3 and F4 dataset using a mixed effects model.

Weighted SNP scores	beta	se	*P*-value	Explained variance in %
Kidney function SNP-score (53 SNPs)	−0.4068	0.1008	5.50E-05	0.33%
HDL cholesterol SNP-score (70 SNPs)	0.0246	0.0130	0.0575	0.06%
Triglycerides SNP-score (38 SNPs)	−0.0430	0.0161	0.0078	0.12%

In addition, lead SNPs from the most recent HDL-C (*n =* 70), triglyceride (*n =* 38) and kidney function (*n =* 53) GWAS were selected. The *P*-values of these partially overlapping 142 SNPs were retrieved from our GWA meta-analysis on log-transformed apoA-IV (Supplementary Material, Tables 6–8). Only one SNP was significantly associated with apoA-IV, rs964184 in *APOA1* (*P =* 0.0001), which is included in the HDL-C as well as in the triglyceride SNP list.

The analyses based on the weighted genetic SNP-scores for kidney function, HDL-C and triglycerides in the combined KORA F3 and F4 dataset yielded two significant results. The weighted kidney function SNP-score was significantly and inversely associated with apoA-IV (*P =* 5.5×10^−^^05^). That means apoA-IV concentrations increased with an increasing number of GFR-decreasing SNPs. Furthermore, a greater number of triglyceride-increasing alleles was shown to be associated with lower apoA-IV concentrations (*P =* 0.0078). The association with HDL-C SNPs was not significant but pointed in the opposite direction as expected: the more HDL-C-increasing alleles, the higher were apoA-IV concentrations (*P =* 0.0554).

## Discussion

This study revealed three major findings. First, using genome-wide data from five studies and two independent replication studies we could identify three independent SNPs from two genomic regions (*APOA4* and *KLKB1*), which were significantly associated with apoA-IV concentrations. Second, approximately one third of the phenotypic variability of apoA-IV seems to be genetically regulated. Third, genetic variants that have a significant effect on kidney function and triglyceride concentrations suggest a causal role of these phenotypes on apoA-IV concentrations.

### Genome-wide significant and replicated SNPs in *APOA4* and *KLKB1*

Conditional stepwise regression analysis including all SNPs in the broad *APOA4* gene region (a 1 MB region including the *APOA5-A4-C3-A1* cluster) led to the identification of two SNPs: the lead SNP rs1729407, located between *APOA5* and *APOA4*, and one missense variant (rs5104). So far, the effect of rs5104 has been studied only in some small studies: it was associated with dyslipidemia in Han Chinese (25), with postprandial ApoA-I plasma concentration in healthy young men (26) and with triglyceride response to fenofibrate treatment (27). Conversely, no association between the lead SNP rs1729407 and any phenotype had been shown until now. The effect of other missense variants in *APOA4* (rs675, rs5110), although widely studied before, could not be replicated. However, these previous studies were markedly smaller, showed contradictory results and investigated different inheritance models (19,20,23).

Both *APOA4* top hits do not present overt functional effects. The lead SNP rs1729407 is located in an intergenic region (Supplementary Material, Figure 10A) while rs5104 causes a serine to asparagine substitution (Ser147Asn), which is classified as benign by common bioinformatics prediction tools. Of note, the lead SNP is in perfect LD (r^2 ^=^ ^1) with a SNP located in a large cluster of transcription factor binding sites located approximately 1.5 kb downstream (rs1729405, *P =* 9.92E-40 in our meta-analysis; Supplementary Material, Figure 10B).

Besides the *APOA4* gene, we also identified a locus on chromosome 4 encompassing the three genes *CYP4V2*, *KLKB1* and *F11*. The top hit was in nearly perfect LD with the missense variant rs3733402 in *KLKB1. KLKB1* encodes the glycoprotein plasma kallikrein (also known “Fletcher factor” (28)), which acts as a proteolytic activator of several vasoactive and circulating peptides (kinins) (29,30). Accordingly, SNPs in *KLKB1* showed genome-wide associations with vasoactive peptides (plasma bradykinin (31,32), active renin (rs3733402) (33), BNP in Blacks (34), aldosterone/renin ratio in Europeans (34), MR-pro-ADM and CT-pro-ET-1 (rs4253238 (35), r^2 ^=^ ^0.81 with our tophit). Of note, *F11* is a paralog of *KLKB1* and codes for the coagulation factor XI. Both are part of the intrinsic pathway (36). However, to our knowledge a mechanism which obviously links apoA-IV to the kinin-kallikrein system or the intrinsic pathway has not been described so far. Therefore, replication and functional studies will be required to appraise the significance of this finding. The third gene in the locus, *CYP4V2, is* a nearly ubiquitously expressed omega-hydroxylase, with the phenotype of loss-of-function mutations being restricted to the eye (37,38) and causing the degenerative ocular disease BCD (39) (OMIM #210370).

Finally, gene-based analysis or pathway-based analysis did not reveal additional novel genes beyond those located in the genomic regions around *APOA4* and *KLKB1*. Since the stepwise conditional analysis resulted in only two independent SNPs located at the *APOA4* or *KLKB1* loci, the observation in the gene-based analysis that multiple genes were significant in each locus could most likely be explained by LD.

### Variance explained and heritability

Another aim of this study was the estimation of the heritability of apoA-IV as well as the variation of apoA-IV explained by all included additive-coded SNPs. Both, genomic and also narrow-sense heritability were calculated to be around 30%. Only a relatively small fraction is explained by the two gene regions we have identified which leaves sufficient room for the discovery of other gene regions. In addition, the major extent of apoA-IV concentrations seems to be regulated by non-genetic factors.

### SNP look-up using results from other GWAS consortia

Another aspect of this study was the look-up of the identified SNPs in other GWAS consortia. Since variants in the *APOA5-A4-C3-A1* gene cluster have consistently been found to be associated with triglycerides and HDL-C (24,40,41), results from the most recent lipids-GWA meta-analysis (24) was used for this look-up. The lead *APOA4*-SNP rs1729407 showed an association with HDL-C (*P =* 7.1×10^−^^07^). However, this SNP seems to be independent from the lead SNP of the lipid-GWA within that gene region (rs964184, reported gene *APOA1*, r^2^ with rs1729407 < 0.1), which had a *P*-value of 6.00E-48 in the GWAS on HDL-C and 7.00×10^−^^224^ in the GWAS on triglycerides (24). SNP rs964184 has also been associated with coronary heart disease on a genome-wide scale, an association possibly triggered by the strong association of rs964184 with triglyceride concentrations (42–44). In our analysis, rs964184 was also associated with apoA-IV (*P =* 0.0001). However, this is far away from genome-wide significance. Altogether, it seems that, despite being within the *APOA5-A4-C3-A1* gene cluster, the SNPs associated with HDL-C and triglycerides are statistically independent from the *APOA4*-SNPs associated with apoA-IV concentrations.

We also performed a look-up to check whether the SNPs detected in our apoA-IV GWAS study were associated with kidney function, defined by eGFR using data from the CKDGen consortium (45). This consortium was chosen because of the already known association of apoA-IV with kidney function and chronic kidney disease (13–16,46). However, none of the *APOA4* and *KLKB1* lead SNPs showed significant associations with eGFR.

We further applied a look-up approach the other way around: when we selected in total 142 unique SNPs that were retrieved from the kidney- and lipid-GWAS, no single SNP was associated with apoA-IV in our GWAS besides the aforementioned SNP in *APOA1* (TG and HDL-C). However, taken together as weighted SNP-scores, the strongest associations with apoA-IV could be found for the kidney-SNP-score and still significant associations for the triglycerides-SNP-score. These results potentially support a possible causal effect of kidney function on apoA-IV concentrations. This might also be true for a potential causal effect of triglycerides on apoA-IV, but to a lesser extent.

So far, only few studies investigated the association between apoA-IV and triglyceride levels, and the results have been inconsistent: for example, no association could be found in the EARS study (1261 controls and 629 cases) (23), whereas a study conducted in 105 participants reported a significantly positive association between apoA-IV and triglyceride levels (47) concentrations. This finding is contradictory to the direction of correlation we found using a triglyceride-increasing SNP-score.

As part of the HDL particle, apoA-IV plays a role as a mediator in the reverse-cholesterol transport (48). Some epidemiological studies also suggest an association of HDL-C with apoA-IV (23). However, a causal role of HDL-C on apoA-IV could not be shown with our data, but also not ruled out. In Hanniman et al. (49), *APOA4* knockout mice showed decreased HDL-C values, whereas overexpression of *APOA4* led to increase of HDL-C, which suggests a causal role of apoA-IV on HDL-C.

## Conclusion

Using data from five population-based studies and two additional replication studies, two independent SNPs located in or next to the *APOA4* gene and one SNP in *KLKB1* gene were significantly associated with apoA-IV levels. These two gene regions alone can only explain a small fraction of the genome-wide explained variance by SNPs which we estimated to be roughly 30%. Therefore, a major part of apoA-IV variability is likely to be regulated by non-genetic factors. Analyses of SNP-scores explaining kidney function, HDL-C and triglyceride levels indicate a potential causal effect of the primary kidney function and by a lesser extent triglycerides on apoA-IV levels.

## Methods

### Study design

The genome-wide SNP association analysis on apoA-IV is based on a two-stage design with a discovery stage and a replication stage (Figure 1). Genome-wide SNP arrays were available for 5 studies of European ancestry (*n =* 13,813 in total). All independent SNPs and missense variants with a *P*-value below 1×10 ^−^ ^6^ were taken forward to the replication stage. In addition, one non-synonymous SNP from the *APOA4* gene that did not fulfill the *P*-value selection criteria was selected for replication (rs675), since it has been widely studied before (18). Altogether, 8 SNPs were then genotyped in both replication studies. Replication of SNPs was achieved, if the following criteria were met: genome-wide significance (*P*  < 5×10 ^−^ ^8^) in the meta-analysis of all 7 studies within the discovery + replication stage (*n =* 16,080 in total), direction of effects in replication studies consistent with the discovery stage and a false-discovery rate (FDR) (50) less than 0.05 on the replication stage.

### GWAS discovery stage: study population, genotyping and imputation

Details on genotyping and imputation for each study can be found in Supplementary Material, Table 2.

The **CoLaus** study is a single-centre, cross-sectional study including 6,182 Caucasian subjects aged 35–75 years from the city of Lausanne in Switzerland (51). From 5,435 participants, genotypes were imputed using the software minimac (52) and 1000 Genomes (phase 1, version 3), resulting in over 7 million SNPs after filtering. Full phenotype information as well as imputed genotypes are available for *n =* 3,996 participants.

For the **NHLBI Family Heart Study (FamHS),** 1,200 families (∼6,000 individuals) were ascertained in 1992, half randomly sampled, half selected because of an excess of coronary heart disease (CHD) or risk factor abnormalities (53). Study participants belonging to the largest pedigrees were invited for a second clinical exam (2002–2004). GWAS analysis was undertaken for 4135 European American subjects using Illumina arrays. SNP genotypes were subsequently imputed with the software MACH (version1.0.16) (54) using 1000 genomes phase 1 version 3 (55) as reference, leading to a total of ∼7.7 million SNPs after filtering. Both imputed genotype data as well as phenotype information was available for *n =* 1,712 participants.

The **KORA F3** study, conducted in the years 2004/05, is a population-based sample from the general population living in the region of Augsburg, Southern Germany, which has evolved from the WHO MONICA study (Monitoring of Trends and Determinants of Cardiovascular Disease). Genome-wide data are available for all participants (*n =* 3,075 with complete phenotype information) based on llumina Omni 2.5/Illumina Omni Express. The **KORA F4** survey is an independent non-overlapping sample drawn from the same population in the years 2006/08 (*n =* 2,926 with complete phenotype information). Genome-wide data are available for all participants in the KORA F4 study (Affymetrix Axiom) (40,56). Both genome-wide genotype data have been imputed with the software IMPUTE using 1000 Genomes phase 1, version 3 (55). After quality control and filtering, about 8.5 M SNPs are available for analyses in both KORA F3 and F4.

The **Cardiovascular Risk in Young Finns Study (YFS)** is a prospective multicenter study from Finland initiated in 1980 (Baseline age 3–18 years) with several follow-ups over 30 years to investigate childhood risk factors for cardiometabolic outcomes (57). For 2443 participants from the 2001 follow-up (ages 24-39 years), high throughput genome wide SNP genotyping using the genome wide Illumina 670K SNP chip was performed at the Wellcome Trust Sanger Centre. Imputation was performed using IMPUTE and the 1000 Genomes Project March 2012 version (phase 1, version 3) as reference, leading to a total of 8.5 million imputed genotypes after filtering. Full phenotype information as well as imputed genotypes is available for *n =* 2,104 participants.

### Replication stage: study population and *de-novo* genotyping

The **Bruneck** study is a prospective population-based survey designed to investigate the epidemiology and pathogenesis of atherosclerosis (58,59). The study population was recruited in 1990 as a sex- and age-stratified random sample of all inhabitants of Bruneck, Italy. The attendance rate was 93.6% with complete data in 919 subjects. An intensive phenotyping was done and follow-up data are available for a period of 25 years.

The **SAPHIR** study (Salzburg Atherosclerosis Prevention Program in subjects at High Individual Risk) is an observational study conducted in the years 1999–2002 involving 1,770 unrelated subjects from a healthy working population. Study participants were recruited by health-screening programs in companies in and around the Austrian city of Salzburg (60). Full phenotype and genotype information is available for *n =* 1,454 participants.

In both studies, de-novo genotyping was performed in a multiplex approach using the SEQUENOM MassArray platform and iPLEX Gold chemistry. Full phenotype and genotype information is available for *n =* 802 participants.

### Measurement of apoA-IV

For all participating studies, quantification of plasma apoA-IV was done in the same laboratory (Division of Genetic Epidemiology, Medical University of Innsbruck, Austria). It was based on a double-antibody enzyme-linked immunosorbent assay using an affinity-purified polyclonal rabbit anti-human apoA-IV antibody for coating and the same antibody coupled to horseradish peroxidase for detection. Plasma with a known concentration of apoA-IV was used as the calibration standard (61). Four control sera with different concentrations were run on each plate in double measurements for control purposes throughout the entire project. The intra- and interassay coefficients of variation were 2.7% and 6.0%, respectively (61).

### Statistical methods

#### GWAS analysis of single studies & discovery stage meta-analysis

An overview of the quality control and meta-analysis workflow in the discovery stage is given in Supplementary Material, Figure 1. Due to the skewed distribution of apoA-IV concentrations, log-transformation of values was performed in all studies, resulting in nearly normal distributions (Supplementary Material, Figure 2). In each study, each SNP was associated with log-transformed apoA-IV concentrations in an additive genetic model using linear regression, adjusted for age and sex. Additionally, linear regression was performed on the untransformed apoA-IV levels to obtain interpretable effect estimates. Since women have slightly lower apoA-IV levels than men, gender-stratified models have also been applied in all studies (62). Genome-wide analysis in the FamHS study was performed using a linear mixed model accounting for familial dependencies described by a pedigree-based kinship matrix.

Quality control and filtering of SNPs was performed centrally and standardized by the Innsbruck study group using EasyQC (63). SNPs were only included in the analysis if they fulfilled the following criteria: imputation quality ≥0.4 (e.g. IMPUTE info), minor allele frequency ≥1% and a *P*-value of the HWE-test ≥ 1×10^−^^06^. Additional analyses for quality control were applied on the already filtered datasets, which included a P-Z-plot (63) and calculation of genomic inflation factor λ. The P-Z-plot compares the reported *P*-values from each study with the *P*-values calculated from Z-statistics derived from the reported beta coefficient and standard error.

For the meta-analysis over all GWAS studies, METASOFT (64) was used for all imputed SNPs that met imputation and quality control criteria. SNPs were only included in the meta-analysis if they were available in 3 or more studies. Based on the heterogeneity between studies for each SNP, a fixed effects (FE) or optimized random effects model (RE) as proposed by (64), was used as implemented in METASOFT. The test statistic for this optimized RE model is partitioned into a mean effects and heterogeneity part. To give higher weights to the mean effects, this RE model was only used when the test statistic for the mean effects part was higher than the heterogeneity part and if the test for heterogeneity was significant (p value of Q statistic < 0.1 & *I*^2 ^≥^ ^50). The test statistics were corrected for genomic inflation in both the GWAS analysis stage and meta-analysis stage. Based on the gender-stratified analyses, a *t*-test on effect differences between men and women was performed (62). All regional plots presenting the *P*-values and LD between SNPs in predefined genomic regions were done using LocusZoom (65).

### SNP selection for replication

To detect independently associated SNPs, a conditional stepwise analysis was performed using the program GCTA (version 1.24.7 (66)). For each locus with at least one *P*-value < 10^−^^6^, the SNP with the lowest *P*-value on the discovery stage was taken as the lead SNP. All SNPs within a region +/- 500 kB surrounding the lead SNP were included in the conditional analysis. GCTA uses the summary-level statistics of the meta-analysis plus one reference population for LD calculation. As reference population, a combined genotype dataset of KORA F3 and KORA F4 was used (*n =* 6,001). By default, the lead SNP is included in the model. Then, all SNPs in the included gene region are tested for association in addition to the already included SNPs. Finally, all SNPs within a gene region with a *P*-value of < 10 ^−^ ^6^ in the conditional analysis were taken forward for replication. Furthermore, all missense mutations with *P*-values of <10^−^^6^ were selected for replication, irrespective of possible LD with already selected SNPs.

### Two-stage meta-analysis

All genotyped SNPs in the replication phase were meta-analyzed in both replication studies separately as well as in a combined analysis of all GWAS and replication-stage studies. Again, METASOFT (64) was used in the same way as in the first stage meta-analysis.

### Gene-based test and pathway analysis

In addition to the analysis of single SNP effects, a gene-based scan and a pathway analysis were performed using KGG version 3.5 (67). Gene regions were defined as the gene ± 20 kb according to the RefGene database. Using this definition, 66.35% of the available SNPs were included in the analysis. For the gene-based analysis, the extended Simes test (GATES) was used as implemented in KGG (68). To adjust for multiple testing, the Bonferroni-method was applied on the number of tested genes. To calculate LD between the SNPs, the 1000G Phase 1 v3 Reference was used. All pathways that are available in the C2 curated gene set from GSEA (http://software.broadinstitute.org/gsea/msigdb/) were included in the pathway analysis. To test for enrichment of each pathway with significant genes, a hypergeometric test as implemented in KGG was used (69). To adjust for multiple testing, the Bonferroni-method was applied on the number of pathways tested.

### Variance explained

The percentage of explained variance for the SNPs that were taken forward for replication was calculated in the SAPHIR study (*n =* 1,465) - as an independent replication cohort - as well as in a combined dataset of both KORA studies (*n =* 6,001). The combined KORA dataset was also used to get an estimate of the proportion of phenotypic variance explained by the regression on additively coded SNPs for a) all SNPs within the *APOA4* and *KLKB1* gene regions, defined as the lead SNPs +/- 500 kB, as in the conditional analysis and b) all available genome-wide imputed SNPs. The latter has been denoted as the genomic heritability (70). Hence, this genomic heritability includes solely the variance attributable to the measured SNP effects. For these analyses, the software GCTA version 1.24.7 was used (66). In the FamHS study, an estimate of the proportion of the additive (polygenic) variance on the phenotypic variance, the narrow-sense heritability h^2^, was obtained using GenABEL's polygenic function, taking the kinship matrix into account. This narrow-sense heritability thus also includes the variance explained by not measured SNPs and other variants (e.g. copy-number-variations). All estimates for the explained variance and heritability refer to log-transformed values of apoA-IV.

### SNP look-up

We performed a look-up of our replicated SNPs in downloadable GWA meta-analysis results on kidney function (defined by eGFR) (71), HDL-C and triglycerides (24). We further looked up lead SNPs identified in these consortia in our apoA-IV GWA meta-analysis. 53 SNPs associated with kidney function, defined by eGFR, were derived from the CKDGen-GWA meta-analysis and 70 SNPs with HDL-C and 38 with triglycerides from the GLGC-GWA meta-analysis. For these SNPs, their respective *P*-values from the log-transformed analysis on apoA-IV levels on the discovery stage were looked up. Altogether, 143 unique SNPs were included in this analysis, some of them involved in more than one phenotype (especially for HDL-C and triglycerides). Therefore, results are declared significant, when the *P*-value is lower than 0.05/143 = 0.00035. Since the effect of single SNPs (and therefore the statistical power) is assumed to be low, we also used the imputed genotypes in both KORA studies to create SNP-scores. Weighting and direction of effects were based on the original publication where the SNPs were derived from. All SNPs were scaled in such a way that they are phenotype and/or risk increasing and weighted by the beta-estimate derived from the respective original study. These weighted genotype scores were then summed up to derive a genetic risk score for each of the phenotypes studied. For these analyses, a combined dataset of KORAF3 and KORAF4 was used (*n =* 6,001). A mixed effects model was performed for this analysis with the study included as a random effects variable. Since three SNP-scores were evaluated, the significance threshold was set to 0.05/3 = 0.0167 for these analyses.

### Bioinformatic analysis

Bioinformatic analysis of intergenic variants using ENCODE data was carried out as described before (72). Analysis of the coding variants was performed using tools Polyphen-2 (73), SIFT (74) and MutPred (75). Pairwise LDs were calculated using SNiPA (76) (http://snipa.helmholtz-muenchen.de) using the European 1000 Genomes Phase 3, v5 dataset.

## Supplementary Material

Supplementary Material is available at *HMG* Online.

Supplementary Data
