# Supplementary material for: A genome-wide association meta-analysis on apolipoprotein A-IV concentrations
Source: Hum Mol Genet. 2016 Jul 12;25(16):3635–46. doi: 10.1093/hmg/ddw211 (PMC5179953; doi:10.1093/hmg/ddw211)
Supplement: Supplementary Data [file supp_25_16_3635__index.html]

A genome-wide association meta-analysis on apolipoprotein A-IV concentrations — A genome-wide association meta-analysis on apolipoprotein A-IV concentrations — Supplementary Data 

# A genome-wide association meta-analysis on apolipoprotein A-IV concentrations

## Supplementary Data

files

- Supplementary Data - pdf file
