# Supplementary material for: A genome-wide association meta-analysis on apolipoprotein A-IV concentrations
Source: Hum Mol Genet. 2016 Jul 12;25(16):3635–46. doi: 10.1093/hmg/ddw211 (PMC5179953; doi:10.1093/hmg/ddw211)
Supplement: Supplementary Data [file supp_ddw211_GWA_apoA4_Supplement_Final.pdf]

## Supplementary Material to

# A Genome-wide Association Meta-analysis on Apolipoprotein A-IV Concentrations


Claudia Lamina [1§], Salome Friedel [1§], Stefan Coassin [1], Rico Rueedi [2,3], Noha A. Yousri [4,5], Ilkka Seppälä [6], Christian Gieger [7,8,9], Sebastian Schönherr [1], Lukas Forer [1], Gertraud Erhart [1], Barbara Kollerits [1], Pedro Marques-Vidal [10], Janina Ried [7], Gerard Waeber [10], Sven Bergmann [2,3], Doreen Dähnhardt [1], Andrea Stöckl [1], Stefan Kiechl [11], Olli T Raitakari [12,13], Mika Kähönen [14], Johann Willeit [11], Ludmilla Kedenko [15], Bernhard Paulweber [15], Annette Peters [8,16,17], Thomas Meitinger [18,19,20], Konstantin Strauch [7,21], KORA study group, Terho Lehtimäki [6], Steven C. Hunt [22,23], Peter Vollenweider [10], Florian Kronenberg [1]*

**[§] *The authors wish it to be known that, in their opinion, the first two authors should be regarded as joint First Authors.***

[1] Division of Genetic Epidemiology, Department of Medical Genetics, Molecular and Clinical Pharmacology, Medical University of Innsbruck, 6020 Innsbruck, Austria
[2] Department of Computational Biology, University of Lausanne, 1015 Lausanne, Switzerland
[3] Swiss Institute of Bioinformatics, 1015 Lausanne, Switzerland
[4] Department of Physiology and Biophysics, Weill Cornell Medical College − Qatar, Doha, Qatar
[5] Department of Computer and Systems Engineering, Alexandria University, 21526 Alexandria, Egypt
[6] Department of Clinical Chemistry, Fimlab Laboratories and University of Tampere School of Medicine, 33520 Tampere, Finland
[7] Institute of Genetic Epidemiology, Helmholtz Zentrum München—German Research Center for Environmental Health, 85764 Neuherberg, Germany
[8] Institute of Epidemiology II, Helmholtz Zentrum München—German Research Center for Environmental Health, 85764 Neuherberg, Germany
[9] Research Unit of Molecular Epidemiology, Helmholtz Zentrum München—German Research Center for Environmental Health, 85764 Neuherberg, Germany
[10] Department of Medicine, Internal Medicine, Centre Hospitalier Universitaire Vaudois (CHUV), 1015 Lausanne, Switzerland
[11] Department of Neurology, Medical University of Innsbruck, 6020 Innsbruck, Austria
[12] Department of Clinical Physiology, Turku University Hospital, 20520 Turku, Finland
[13] Research Centre of Applied and Preventive Cardiovascular Medicine, University of Turku, 20520 Turku, Finland
[14] Department of Clinical Physiology, Tampere University Hospital and University of Tampere, 33521 Tampere, Finland
[15] First Department of Internal Medicine, Paracelsus Private Medical University, 5020 Salzburg, Austria
[16] DZHK (German Centre for Cardiovascular Research), partner site Munich Heart Alliance, 80802 Munich, Germany
[17] German Center for Diabetes Research (DZD e.V.), 85764 Neuherberg, Germany
[18] Institute of Human Genetics, Technische Universität München, 81675 München, Germany
[19] Institute of Human Genetics, Helmholtz Zentrum München, 85764 Neuherberg, Germany
[20] Munich Cluster for Systems Neurology (SyNergy), 81377 Munich, Germany
[21] Institute of Medical Informatics, Biometry and Epidemiology, Chair of Genetic Epidemiology, Ludwig-Maximilians-Universität, 81377 Munich, Germany
[22] Cardiovascular Genetics Division, University of Utah School of Medicine, Salt Lake City, UT 84108, USA
[23] Department of Genetic Medicine, Weill Cornell Medicine, Doha, Qatar

***Address of correspondence:**
Florian Kronenberg, MD
Division of Genetic Epidemiology
Department of Medical Genetics, Molecular and Clinical Pharmacology
Innsbruck Medical University
Schöpfstr. 41, A-6020 Innsbruck, AUSTRIA
Phone: (+43) 512 9003-70560
Fax: (+43) 512 9003-73560 or -73561
E-mail: Florian.Kronenberg@i-med.ac.at

## Supplementary Tables

**Supplementary Table 1:** Baseline characteristics of contributing studies

| | Discovery stage | | | | | Replication stage | |
|---|---|---|---|---|---|---|---|
| | CoLaus (n=3996) | FamHS (n=1712) | KORA F3 (n=3075) | KORA F4 (n=2926) | YFS (n=2104) | Bruneck (n=802) | SAPHIR (n=1465) |
| Age (in years) | 58.54 ± 10.52 [50, 58, 67] | 51.5 ± 14.1 [39, 53, 63] | 57.31 ± 12.89 [46, 57, 67] | 56.15 ± 13.31 [44, 56, 67] | 31.73 ± 4.99 [27, 33, 36] | 62.7 ± 11.1 [53, 62, 71] | 51 ± 6.02 [46, 52, 55] |
| Women, n (%) | 1858 (46.50%) | 1022 (54.0%) | 1577 (51.28%) | 1509 (51.57%) | 1156 (54.94%) | 402 (49.26%) | 476 (32.49%) |
| ApoA-IV (mg/dl) | 16.28 ± 4.73 [13.20, 15.77, 18.74] | 14.3 ± 4.1 [11.6, 13.9, 16.6] | 17.25 ± 4.67 [14.16, 16.72, 19.80] | 15.31 ± 4.34 [12.40, 14.95, 17.62] | 13.13 ± 3.84 [10.44, 12.84, 15.23] | 14.27 ± 3.61 [11.90, 12.20, 16.50] | 14.55 ± 3.3 [12.31, 14.50, 16.60] |
| Triglycerides (mg/dl) | 120.02 ± 77.62 [71, 97, 142] | 149.2 ± 104.4 [84, 125, 185] | 164.66 ± 125.84 [88, 135, 200] | 125.04 ± 89.18 [72, 105, 151] | 118.35 ± 75.69 [71, 97, 142] | 131.52 ± 79.87 [81, 111, 157] | 126.38 ± 89.81 [72.0, 101.5, 151.0] |
| HDL cholesterol (mg/dl) | 63.59 ± 18.02 [50, 62, 73] | 49.8 ± 15.0 [39, 48, 58] | 58.85 ± 17.14 [47, 56, 69] | 55.91 ± 14.45 [45, 54, 65] | 49.83 ± 12.23 [41, 49, 58] | 58.75 ± 16.13 [47, 57, 67] | 59.19 ± 15.6 [48, 57, 68] |
| eGFR (CKD-EPI) (mL/min/1.73 m2) | 81.93 ± 14.87 [72.17, 82.53, 92.61] | 81.7 ± 16.7 [71.0, 81.4, 93.2] | 82.73 ± 17.61 [71.55, 84.43, 95.26] | 83.76 ± 17.15 [72.72, 84.84, 96.05] | 114.11 ± 6.45 [109.80, 113.80, 118.46] | 79.24 ± 15.03 [69.35, 80.61, 89.66] | 95.46 ± 11.83 [86.24, 98.54, 104.16] |

Continuous variables are shown as mean +/- stdev and [25%, 50%, 75%]-Percentiles

**Supplementary Table 2:** Genotyping and imputation information of contributing studies at the discovery stage

| | CoLaus | FamHS | KORA F3 | KORA F4 | YFS |
|---|---|---|---|---|---|
| SNP-chip | Affymetrix GeneChip® Human Mapping 500K | Illumina Human 1M-Duo v3<br><br>Illumina 610-Quadv1<br><br>Illumina Human Hap550K | Illumina Omni 2.5/<br><br>Illumina Omni Express | Affymetrix Axiom | Illumina 670k |
| QC before imputation | callrate >70%, p-value HWE > $10^{-7}$ | callrate >98%, p-value HWE > $10^{-6}$, MAF>1% | callrate >98%, p-value HWE > $10^{-10}$ | callrate >98%, p-value HWE > $10^{-10}$ | callrate >95%, p-value HWE > $10^{-6}$, MAF>1% |
| Program used for Imputation | minimac | MACH | IMPUTE | IMPUTE | IMPUTE |
| Reference panel for imputation | 1000G phase 1 v3 | 1000G phase 1 v3 | 1000G phase 1 v3 | 1000G phase 1 v3 | 1000G phase 1 v3 |
| Program used for GWA analysis | In-house Matlab code | PROBABEL | SNPTest | SNPTest | SNPTest 2.4.1 |
| Genomic inflation factor $\lambda$ | 1.038 | 1.015 | 1.023 | 1.011 | 1.014 |

**Supplementary Table 3:** Characteristics of selected SNPs for replication (p-value < 1E-06 in the GWAs stage)

| SNP | Chr | Position[#] | Nearest Gene | a* | A* | Reason for selection | MAF (range in studies) | MAF (1000G EUR)* |
|---|---|---|---|---|---|---|---|---|
| **APOA4 gene region** | | | | | | | | |
| rs1729407 | 11 | 116677370 | *APOA4* | C | G | Lead SNP | 0.485-0.546 | 0.498 |
| rs5104 | 11 | 116692334 | *APOA4* | C | T | Missense variant & independently associated (cond. analysis) | 0.128-0.204 | 0.174 |
| rs5110 | 11 | 116691634 | *APOA4* | A | C | Missense variant & Known from literature | 0.035- 0.080 | 0.066 |
| **APOA4 gene region, selected from literature** | | | | | | | | |
| rs675 | 11 | 116691675 | *APOA4* | A (C) | T | Missense variant & known from literature | 0.158-0.209 | 0.180 (<0.001) |
| **Other gene regions** | | | | | | | | |
| rs4241819 | 4 | 187157140 | *KLKB1* | C | T | Lead SNP, but could not be genotyped → used in GWAs stage | 0.452-0.565[$] | 0.478 |
| rs4253311 | 4 | 187174683 | *KLKB1* | A | G | Proxy SNP with lead SNP and with missense variant → used in replication stage | | 0.477 |
| rs59698941 | 5 | 132154912 | *SOWAHA* | G | A | Lead SNP | 0.140-0.167 | 0.127 |
| rs2292030 | 5 | 132161294 | *SHROOM1* | A | G | Missense variant | 0.138-0.160 | 0.120 |

* MAF, minor (a) and major alleles (A) derived from 1000 Genomes, phase 3 v5, all European populations
[$] In GWAs discovery stage studies; [#]Based on build 37, HG19

**Supplementary Table 4:** Study-specific results of SNPs that were selected for replication

| | MAF | HWE | Info* | Call-rate** | $\beta^{\#}$ | $se^{\#}$ | $p^{\#}$ | $\beta^{\&}$ | $se^{\&}$ | $p^{\&}$ |
|---|---|---|---|---|---|---|---|---|---|---|
| **rs1729407** | | | | | | | | | | |
| CoLaus | 0.510 | 0.884 | 0.892 | -- | -0.8440 | 0.1053 | 1.11E-15 | -0.0509 | 0.0064 | 1.39E-15 |
| FamHS | 0.485 | 0.685 | 0.778 | -- | -0.5303 | 0.1473 | 3.16E-04 | -0.0397 | 0.0104 | 1.29E-04 |
| KORA F3 | 0.509 | 0.248 | 0.975 | -- | -0.7803 | 0.1187 | 5.68E-11 | -0.0452 | 0.0068 | 3.55E-11 |
| KORA F4 | 0.498 | 0.579 | 0.977 | -- | -0.7970 | 0.1102 | 5.95E-13 | -0.0527 | 0.0071 | 1.14E-13 |
| YFS | 0.546 | 0.291 | 0.924 | -- | -0.0904 | 0.0326 | 0.0056 | -0.0951 | 0.0326 | 0.0035 |
| Bruneck | 0.510 | 0.926 | -- | 0.988 | -0.3382 | 0.1808 | 0.0617 | -0.0209 | 0.0135 | 0.1231 |
| SAPHIR | 0.510 | 0.949 | -- | 0.992 | -0.5568 | 0.1206 | 4.24E-06 | -0.0394 | 0.0087 | 6.95e-06 |
| **rs5104** | | | | | | | | | | |
| CoLaus | 0.151 | 0.477 | 0.965 | -- | -0.6960 | 0.1414 | 8.54E-07 | -0.0425 | 0.0086 | 6.81E-07 |
| FamHS | 0.128 | 0.944 | 0.713 | -- | -0.5951 | 0.2148 | 0.0056 | -0.0383 | 0.0151 | 0.0113 |
| KORA F3 | 0.140 | 0.084 | 0.979 | -- | -0.9772 | 0.1719 | 1.43E-08 | -0.0557 | 0.0099 | 1.75E-08 |
| KORA F4 | 0.144 | 0.598 | 0.947 | -- | -0.8742 | 0.1601 | 5.14E-08 | -0.0602 | 0.0103 | 5.08E-09 |
| YFS | 0.204 | 0.503 | 0.885 | -- | -0.1130 | 0.0402 | 0.0050 | -0.1105 | 0.0402 | 0.0061 |
| Bruneck | 0.109 | 0.605 | -- | 0.984 | -0.4327 | 0.2889 | 0.1346 | -0.0341 | 0.0216 | 0.1153 |
| SAPHIR | 0.143 | 0.157 | -- | 0.986 | -0.4603 | 0.1692 | 0.0066 | -0.0344 | 0.01223 | 0.0049 |
| **rs5110** | | | | | | | | | | |
| CoLaus | 0.072 | 0.849 | 0.517 | -- | 0.8257 | 0.2663 | 0.0019 | 0.0432 | 0.0161 | 0.0074 |
| FamHS | 0.080 | 0.274 | 0.631 | -- | 0.5338 | 0.2980 | 0.0732 | 0.0385 | 0.0209 | 0.0658 |
| KORA F3 | 0.074 | 0.293 | 1.000 | -- | 0.5140 | 0.2201 | 0.0196 | 0.0342 | 0.0126 | 0.0068 |
| KORA F4 | 0.065 | 0.541 | 0.771 | -- | 0.6306 | 0.2531 | 0.0128 | 0.0442 | 0.0162 | 0.0065 |
| YFS | 0.035 | 1.000 | 0.787 | -- | 0.0182 | 0.0953 | 0.8484 | 0.0405 | 0.0953 | 0.6711 |
| Bruneck | 0.082 | 0.482 | -- | 0.995 | -0.1301 | 0.3214 | 0.6857 | -0.0122 | 0.0240 | 0.6121 |
| SAPHIR | 0.067 | 0.193 | -- | 0.994 | 0.1591 | 0.2465 | 0.5188 | 0.0092 | 0.0179 | 0.6066 |
| **rs675** | | | | | | | | | | |
| CoLaus | 0.197 | 0.497 | 0.969 | -- | -0.2894 | 0.1282 | 0.0240 | -0.0187 | 0.0078 | 0.0156 |
| FamHS | 0.207 | 0.026 | 0.842 | -- | -0.1456 | 0.1822 | 0.4244 | -0.0141 | 0.0128 | 0.2725 |
| KORA F3 | 0.208 | 0.353 | 0.997 | -- | -0.1200 | 0.1426 | 0.4001 | -0.0069 | 0.0082 | 0.3977 |
| KORA F4 | 0.209 | 0.313 | 0.923 | -- | -0.2091 | 0.1399 | 0.1351 | -0.0143 | 0.0090 | 0.1119 |
| YFS | 0.158 | 0.935 | 0.932 | -- | -0.0693 | 0.0439 | 0.1147 | -0.0625 | 0.0439 | 0.1546 |
| Bruneck | 0.249 | 0.700 | -- | 0.999 | -0.0838 | 0.2051 | 0.6830 | -0.0025 | 0.0154 | 0.8692 |
| SAPHIR | 0.228 | 0.754 | -- | 0.991 | -0.1774 | 0.1449 | 0.2210 | -0.0122 | 0.0105 | 0.2458 |
| **rs4241819 in GWAs stage / rs4253311 in replication stage** | | | | | | | | | | |
| CoLaus | 0.540 | 0.975 | 0.787 | -- | 0.4756 | 0.1138 | 2.92E-05 | 0.0307 | 0.0069 | 7.87e-06 |
| FamHS | 0.534 | 0.513 | 0.884 | -- | 0.2440 | 0.1464 | 0.0955 | 0.0187 | 0.0103 | 0.0688 |
| KORA F3 | 0.565 | 0.741 | 0.935 | -- | 0.3102 | 0.1212 | 0.0105 | 0.0183 | 0.0070 | 0.0083 |
| KORA F4 | 0.553 | 0.370 | 0.932 | -- | 0.5290 | 0.1136 | 3.36E-06 | 0.0343 | 0.0073 | 2.63e-06 |
| YFS | 0.452 | 0.965 | 0.937 | -- | 0.0728 | 0.0320 | 0.0229 | 0.0920 | 0.0320 | 0.0040 |
| Bruneck | 0.530 | 0.973 | -- | 0.988 | 0.3800 | 0.1810 | 0.0362 | 0.0307 | 0.0135 | 0.0236 |
| SAPHIR | 0.510 | 0.710 | -- | 0.993 | 0.1789 | 0.1211 | 0.1397 | 0.0143 | 0.0087 | 0.1032 |
| **rs59698941** | | | | | | | | | | |
| CoLaus | 0.157 | 0.770 | 0.781 | -- | -0.4363 | 0.1555 | 0.0050 | -0.0246 | 0.0094 | 0.0089 |
| FamHS | 0.148 | 0.001 | 0.913 | -- | -0.8484 | 0.2062 | 3.87E-05 | -0.0649 | 0.0145 | 7.58E-06 |
| KORA F3 | 0.155 | 0.581 | 0.994 | -- | -0.1263 | 0.1627 | 0.4375 | -0.0057 | 0.0093 | 0.5423 |
| KORA F4 | 0.167 | 0.894 | 0.894 | -- | -0.4603 | 0.1562 | 0.0032 | -0.0284 | 0.0100 | 0.0047 |
| YFS | 0.140 | 0.928 | 0.959 | -- | -0.0557 | 0.0453 | 0.2183 | -0.0766 | 0.0452 | 0.0907 |
| Bruneck | 0.156 | 0.014 | -- | 0.990 | 0.1860 | 0.2372 | 0.4332 | 0.0107 | 0.0177 | 0.5485 |
| SAPHIR | 0.133 | 0.948 | -- | 0.994 | -0.1488 | 0.1815 | 0.4124 | -0.0138 | 0.0132 | 0.2956 |
| **rs2292030** | | | | | | | | | | |
| CoLaus | 0.138 | 0.470 | 0.877 | -- | -0.4162 | 0.1549 | 0.0072 | -0.0226 | 0.0094 | 0.0160 |
| FamHS | 0.140 | 0.001 | 0.952 | -- | -0.8228 | 0.2081 | 7.71E-05 | -0.0628 | 0.0146 | 1.79E-05 |
| KORA F3 | 0.148 | 0.223 | 0.988 | -- | -0.1339 | 0.1672 | 0.4231 | -0.0056 | 0.0096 | 0.5629 |
| KORA F4 | 0.160 | 0.945 | 0.913 | -- | -0.4801 | 0.1573 | 0.0023 | -0.0296 | 0.0101 | 0.0034 |
| YFS | 0.140 | 1.000 | 0.965 | -- | -0.0540 | 0.0452 | 0.2321 | -0.0750 | 0.0451 | 0.0967 |
| Bruneck | 0.150 | 0.051 | -- | 0.967 | 0.1072 | 0.2461 | 0.6632 | 0.0039 | 0.0184 | 0.8316 |
| SAPHIR | 0.126 | 0.965 | -- | 0.976 | -0.0973 | 0.1874 | 0.6036 | -0.0104 | 0.0136 | 0.4430 |

*Info: imputation quality, for all discovery stage cohorts; **For replication cohorts; #B on original scale of ApoA-IV; & Based on log-transformed ApoA-IV values; all β-estimates and MAF refer to the minor allele (1000G EUR, Table 3)

**Supplementary Table 5:** Results of pathway association analysis with all included genes in each pathway; Pathways are sorted by the Bonferroni-corrected p-value in ascending order.

| Gene set | Gene set p-value (Hypergeometric test, Bonferroni-corrected) | Number of genes in gene set | Gene name | Gene-based p-value | chr | Start | Number of SNPs |
|---|---|---|---|---|---|---|---|
| REACTOME_CHYLOMICRON_MEDIATED_LIPID_TRANSPORT | 0.00000014 | 15 | | | | | |
| | | | APOA5 | 2.14E-38 | 11 | 116660085 | 134 |
| | | | APOC3 | 7.2E-38 | 11 | 116700623 | 173 |
| | | | APOA1 | 1.23E-29 | 11 | 116706468 | 171 |
| | | | P4HB | 0.07675 | 17 | 79801033 | 141 |
| | | | APOB | 0.10523 | 2 | 21224300 | 174 |
| | | | SDC1 | 0.23886 | 2 | 20400557 | 240 |
| | | | APOA2 | 0.32657 | 1 | 161192082 | 112 |
| | | | LPL | 0.45164 | 8 | 19796581 | 293 |
| | | | HSPG2 | 0.48969 | 1 | 22148724 | 548 |
| | | | APOC2 | 0.52755 | 19 | 45449238 | 151 |
| | | | LIPC | 0.54093 | 15 | 58724174 | 659 |
| | | | LDLRAP1 | 0.63487 | 1 | 25870075 | 157 |
| | | | LDLR | 0.73131 | 19 | 11200037 | 338 |
| | | | SAR1B | 0.80991 | 5 | 133936838 | 98 |
| | | | APOE | 0.91052 | 19 | 45409657 | 140 |
| REACTOME_LIPOPROTEIN_METABOLISM | 0.000000893 | 27 | | | | | |
| | | | APOA5 | 2.14E-38 | 11 | 116660085 | 134 |
| | | | APOC3 | 7.2E-38 | 11 | 116700623 | 173 |
| | | | APOA1 | 1.23E-29 | 11 | 116706468 | 171 |
| | | | LCAT | 0.07266 | 16 | 67973786 | 46 |
| | | | P4HB | 0.07675 | 17 | 79801033 | 141 |
| | | | APOB | 0.10523 | 2 | 21224300 | 174 |
| | | | CETP | 0.13017 | 16 | 56995834 | 235 |
| | | | SCARB1 | 0.14549 | 12 | 125262173 | 458 |
| | | | AMN | 0.21923 | 14 | 103388992 | 135 |
| | | | SDC1 | 0.23886 | 2 | 20400557 | 240 |
| | | | BMP1 | 0.2825 | 8 | 22022652 | 291 |
| | | | APOA2 | 0.32657 | 1 | 161192082 | 112 |
| | | | ABCG1 | 0.37514 | 21 | 43640007 | 537 |

| Gene set | Gene set p-value (Hypergeometric test, Bonferroni-corrected) | Number of genes in gene set | Gene name | Gene-based p-value | chr | Start | Number of SNPs |
|---|---|---|---|---|---|---|---|
| | | | LPL | 0.45164 | 8 | 19796581 | 293 |
| | | | CUBN | 0.46678 | 10 | 16865964 | 1440 |
| | | | HSPG2 | 0.48969 | 1 | 22148724 | 548 |
| | | | APOC2 | 0.52755 | 19 | 45449238 | 151 |
| | | | LIPC | 0.54093 | 15 | 58724174 | 659 |
| | | | LDLRAP1 | 0.63487 | 1 | 25870075 | 157 |
| | | | ABCA1 | 0.72544 | 9 | 107543283 | 775 |
| | | | LDLR | 0.73131 | 19 | 11200037 | 338 |
| | | | PLTP | 0.7649 | 20 | 44527258 | 148 |
| | | | SAR1B | 0.80991 | 5 | 133936838 | 98 |
| | | | ALB | 0.8635 | 4 | 74269971 | 121 |
| | | | LPA | 0.89897 | 6 | 160952514 | 346 |
| | | | APOE | 0.91052 | 19 | 45409657 | 140 |
| | | | A2M | 0.96774 | 12 | 9220303 | 194 |
| REACTOME_LIPID_DIGESTION_MOBILIZATION_AND_TRANSPORT | 0.00000401 | 44 | | | | | |
| | | | APOA5 | 2.14E-38 | 11 | 116660085 | 134 |
| | | | APOC3 | 7.2E-38 | 11 | 116700623 | 173 |
| | | | APOA1 | 1.23E-29 | 11 | 116706468 | 171 |
| | | | PNLIPRP2 | 0.0078 | 10 | 118380464 | 247 |
| | | | PRKACB | 0.06885 | 1 | 84647314 | 367 |
| | | | LCAT | 0.07266 | 16 | 67973786 | 46 |
| | | | P4HB | 0.07675 | 17 | 79801033 | 141 |
| | | | LIPE | 0.09676 | 19 | 42905658 | 77 |
| | | | APOB | 0.10523 | 2 | 21224300 | 174 |
| | | | CETP | 0.13017 | 16 | 56995834 | 235 |
| | | | SCARB1 | 0.14549 | 12 | 125262173 | 458 |
| | | | PNLIP | 0.17251 | 10 | 118305427 | 113 |
| | | | MGLL | 0.18105 | 3 | 127407904 | 475 |
| | | | PPP1CC | 0.19497 | 12 | 111157612 | 154 |
| | | | AMN | 0.21923 | 14 | 103388992 | 135 |
| | | | SDC1 | 0.23886 | 2 | 20400557 | 240 |
| | | | BMP1 | 0.2825 | 8 | 22022652 | 291 |

| Gene set | Gene set p-value (Hypergeometric test, Bonferroni-corrected) | Number of genes in gene set | Gene name | Gene-based p-value | chr | Start | Number of SNPs |
|---|---|---|---|---|---|---|---|
| | | | ABCG5 | 0.28784 | 2 | 44039610 | 296 |
| | | | APOA2 | 0.32657 | 1 | 161192082 | 112 |
| | | | ABCG1 | 0.37514 | 21 | 43640007 | 537 |
| | | | LPL | 0.45164 | 8 | 19796581 | 293 |
| | | | CUBN | 0.46678 | 10 | 16865964 | 1440 |
| | | | HSPG2 | 0.48969 | 1 | 22148724 | 548 |
| | | | APOC2 | 0.52755 | 19 | 45449238 | 151 |
| | | | LIPC | 0.54093 | 15 | 58724174 | 659 |
| | | | ABCG8 | 0.56245 | 2 | 44066102 | 447 |
| | | | PLIN1 | 0.58966 | 15 | 90207599 | 162 |
| | | | CLPS | 0.60749 | 6 | 35762758 | 165 |
| | | | LDLRAP1 | 0.63487 | 1 | 25870075 | 157 |
| | | | FABP4 | 0.64051 | 8 | 82390731 | 130 |
| | | | PRKACA | 0.647 | 19 | 14202506 | 96 |
| | | | PRKACG | 0.69531 | 9 | 71627448 | 125 |
| | | | ABCA1 | 0.72544 | 9 | 107543283 | 775 |
| | | | LDLR | 0.73131 | 19 | 11200037 | 338 |
| | | | PLTP | 0.7649 | 20 | 44527258 | 148 |
| | | | SAR1B | 0.80991 | 5 | 133936838 | 98 |
| | | | ALB | 0.8635 | 4 | 74269971 | 121 |
| | | | PPP1CA | 0.8795 | 11 | 67165651 | 79 |
| | | | CAV1 | 0.8919 | 7 | 116166411 | 216 |
| | | | LPA | 0.89897 | 6 | 160952514 | 346 |
| | | | APOE | 0.91052 | 19 | 45409657 | 140 |
| | | | ABHD5 | 0.95474 | 3 | 43732374 | 195 |
| | | | A2M | 0.96774 | 12 | 9220303 | 194 |
| | | | PPP1CB | 0.97427 | 2 | 28974625 | 197 |
| KEGG_PPAR_SIGNALING_PATHWAY | 0.0000144 | 67 | | | | | |
| | | | APOA5 | 2.14E-38 | 11 | 116660085 | 134 |
| | | | APOC3 | 7.2E-38 | 11 | 116700623 | 173 |
| | | | APOA1 | 1.23E-29 | 11 | 116706468 | 171 |
| | | | FABP2 | 0.002 | 4 | 120238404 | 274 |

| Gene set | Gene set p-value (Hypergeometric test, Bonferroni-corrected) | Number of genes in gene set | Gene name | Gene-based p-value | chr | Start | Number of SNPs |
|---|---|---|---|---|---|---|---|
| | | | ACOX2 | 0.01785 | 3 | 58490862 | 174 |
| | | | RXRG | 0.02237 | 1 | 165406164 | 347 |
| | | | CYP8B1 | 0.04114 | 3 | 42913683 | 123 |
| | | | PPARD | 0.04218 | 6 | 35310334 | 362 |
| | | | ACOX1 | 0.06478 | 17 | 73937588 | 248 |
| | | | SCD | 0.06679 | 10 | 102106771 | 147 |
| | | | DBI | 0.08871 | 2 | 120125250 | 188 |
| | | | FABP3 | 0.12868 | 1 | 31838099 | 136 |
| | | | RXRB | 0.14515 | 6 | 33161361 | 123 |
| | | | UBC | 0.15962 | 12 | 125396190 | 142 |
| | | | PPARG | 0.16454 | 3 | 12393000 | 482 |
| | | | SLC27A4 | 0.16824 | 9 | 131102838 | 129 |
| | | | ILK | 0.19735 | 11 | 6625039 | 118 |
| | | | SLC27A1 | 0.19767 | 19 | 17581252 | 259 |
| | | | UCP1 | 0.25521 | 4 | 141481049 | 127 |
| | | | ACSL3 | 0.26547 | 2 | 223725731 | 358 |
| | | | RXRA | 0.27856 | 9 | 137298427 | 491 |
| | | | CYP27A1 | 0.27923 | 2 | 219646471 | 163 |
| | | | MMP1 | 0.3174 | 11 | 102660640 | 189 |
| | | | APOA2 | 0.32657 | 1 | 161192082 | 112 |
| | | | ACSL6 | 0.3356 | 5 | 131285666 | 171 |
| | | | CPT2 | 0.34885 | 1 | 53662100 | 125 |
| | | | ACADM | 0.39328 | 1 | 76190031 | 310 |
| | | | LPL | 0.45164 | 8 | 19796581 | 293 |
| | | | AQP7 | 0.50619 | 9 | 33384947 | 169 |
| | | | OLR1 | 0.51463 | 12 | 10310898 | 222 |
| | | | SLC27A6 | 0.52504 | 5 | 128301212 | 618 |
| | | | FABP7 | 0.53044 | 6 | 123100645 | 140 |
| | | | HMGCS2 | 0.56216 | 1 | 120290618 | 148 |
| | | | PLIN1 | 0.58966 | 15 | 90207599 | 162 |
| | | | SCD5 | 0.63508 | 4 | 83581039 | 1017 |
| | | | FABP4 | 0.64051 | 8 | 82390731 | 130 |

| Gene set | Gene set p-value (Hypergeometric test, Bonferroni-corrected) | Number of genes in gene set | Gene name | Gene-based p-value | chr | Start | Number of SNPs |
|---|---|---|---|---|---|---|---|
| | | | FADS2 | 0.69986 | 11 | 61595504 | 228 |
| | | | FABP5 | 0.71816 | 8 | 82192717 | 69 |
| | | | PCK1 | 0.72845 | 20 | 56136136 | 226 |
| | | | ADIPOQ | 0.7557 | 3 | 186560462 | 193 |
| | | | PLTP | 0.7649 | 20 | 44527258 | 148 |
| | | | PPARA | 0.76737 | 22 | 46546498 | 340 |
| | | | SLC27A5 | 0.81851 | 19 | 59009699 | 114 |
| | | | ACADL | 0.83862 | 2 | 211052715 | 82 |
| | | | GK2 | 0.8464 | 4 | 80327506 | 63 |
| | | | CD36 | 0.86119 | 7 | 80275968 | 350 |
| | | | CPT1C | 0.86906 | 19 | 50194364 | 171 |
| | | | ANGPTL4 | 0.87016 | 19 | 8429010 | 176 |
| | | | ACSL1 | 0.87476 | 4 | 185676748 | 379 |
| | | | NR1H3 | 0.8767 | 11 | 47279467 | 158 |
| | | | FABP1 | 0.89514 | 2 | 88422507 | 153 |
| | | | CPT1A | 0.92095 | 11 | 68522350 | 303 |
| | | | FABP6 | 0.92298 | 5 | 159656436 | 449 |
| | | | SORBS1 | 0.92504 | 10 | 97071529 | 1051 |
| | | | PDPK1 | 0.94277 | 16 | 2587964 | 62 |
| | | | SLC27A2 | 0.94331 | 15 | 50474392 | 394 |
| | | | PCK2 | 0.95745 | 14 | 24563489 | 79 |
| | | | ACAA1 | 0.98709 | 3 | 38164200 | 80 |
| | | | ME1 | 0.98836 | 6 | 83920109 | 615 |
| | | | SCP2 | 0.98858 | 1 | 53480609 | 626 |
| | | | CYP4A11 | 0.99122 | 1 | 47394845 | 152 |
| | | | EHHADH | 0.99302 | 3 | 184908411 | 229 |
| | | | CYP7A1 | 0.99403 | 8 | 59402736 | 127 |
| | | | ACOX3 | 0.9988 | 4 | 8368008 | 513 |
| | | | ACSL5 | 0.99882 | 10 | 114135955 | 206 |
| | | | CPT1B | 0.9994 | 22 | 51007289 | 282 |
| | | | CYP4A22 | 0.99993 | 1 | 47603096 | 175 |
| YAMASHITA_LIVER_CANCER_STEM_CELL_DN | 0.0000186 | 73 | | | | | |

| Gene set | Gene set p-value (Hypergeometric test, Bonferroni-corrected) | Number of genes in gene set | Gene name | Gene-based p-value | chr | Start | Number of SNPs |
|---|---|---|---|---|---|---|---|
| | | | APOA5 | 2.14E-38 | 11 | 116660085 | 134 |
| | | | KLKB1 | 5.86E-11 | 4 | 187148671 | 258 |
| | | | F11 | 9.03E-10 | 4 | 187187117 | 222 |
| | | | FMO3 | 0.00234 | 1 | 171060017 | 277 |
| | | | CYP8B1 | 0.04114 | 3 | 42913683 | 123 |
| | | | C1R | 0.04702 | 12 | 7187514 | 111 |
| | | | SAA4 | 0.06727 | 11 | 18252901 | 184 |
| | | | C8B | 0.09652 | 1 | 57394882 | 375 |
| | | | UGT2B7 | 0.09948 | 4 | 69962192 | 278 |
| | | | SERPINC1 | 0.10496 | 1 | 173872941 | 135 |
| | | | ADCY8 | 0.13823 | 8 | 131792546 | 1117 |
| | | | CYP3A7 | 0.15094 | 7 | 99302659 | 130 |
| | | | GSTP1 | 0.25614 | 11 | 67351065 | 139 |
| | | | ACSL3 | 0.26547 | 2 | 223725731 | 358 |
| | | | ABCG5 | 0.28784 | 2 | 44039610 | 296 |
| | | | SLC10A1 | 0.30116 | 14 | 70242551 | 136 |
| | | | MASP2 | 0.30869 | 1 | 11104854 | 142 |
| | | | SOD1 | 0.31 | 21 | 33031934 | 124 |
| | | | GSTA1 | 0.32154 | 6 | 52656177 | 150 |
| | | | SNAI2 | 0.33088 | 8 | 49830238 | 67 |
| | | | THBS1 | 0.3414 | 15 | 39873279 | 119 |
| | | | MAPK13 | 0.36382 | 6 | 36098260 | 97 |
| | | | NR1I3 | 0.36512 | 1 | 161199455 | 153 |
| | | | CD14 | 0.36598 | 5 | 140011312 | 113 |
| | | | ALDH6A1 | 0.38319 | 14 | 74524367 | 244 |
| | | | ESR1 | 0.4032 | 6 | 152128813 | 1500 |
| | | | CES2 | 0.41484 | 16 | 66968373 | 77 |
| | | | PRKCD | 0.41655 | 3 | 53195536 | 193 |
| | | | CES1 | 0.42738 | 16 | 55836763 | 294 |
| | | | SULT1C4 | 0.43638 | 2 | 108994420 | 150 |
| | | | APCS | 0.44131 | 1 | 159557615 | 179 |
| | | | CRABP1 | 0.48296 | 15 | 78632665 | 120 |

| Gene set | Gene set p-value (Hypergeometric test, Bonferroni-corrected) | Number of genes in gene set | Gene name | Gene-based p-value | chr | Start | Number of SNPs |
|---|---|---|---|---|---|---|---|
| | | | DNAJA4 | 0.48797 | 15 | 78558559 | 204 |
| | | | HNF4A | 0.49621 | 20 | 43029895 | 394 |
| | | | MAP2K2 | 0.54286 | 19 | 4090319 | 234 |
| | | | FKBP5 | 0.55259 | 6 | 35548333 | 473 |
| | | | ABCB4 | 0.55526 | 7 | 87031360 | 267 |
| | | | CYP3A4 | 0.59613 | 7 | 99354582 | 68 |
| | | | HMGCR | 0.64022 | 5 | 74632992 | 162 |
| | | | C8A | 0.64583 | 1 | 57320442 | 377 |
| | | | CYP7B1 | 0.66661 | 8 | 65508528 | 492 |
| | | | FABP5 | 0.71816 | 8 | 82192717 | 69 |
| | | | RARG | 0.74134 | 12 | 53604349 | 125 |
| | | | MAP3K5 | 0.74356 | 6 | 136878186 | 504 |
| | | | APOC4 | 0.76133 | 19 | 45445494 | 147 |
| | | | UGT2B15 | 0.77481 | 4 | 69512314 | 287 |
| | | | MASP1 | 0.81646 | 3 | 186964141 | 380 |
| | | | NR1I2 | 0.84307 | 3 | 119501556 | 214 |
| | | | ALDH8A1 | 0.85237 | 6 | 135238527 | 148 |
| | | | ALDH1L1 | 0.85926 | 3 | 125822403 | 517 |
| | | | PIK3C3 | 0.8607 | 18 | 39535162 | 286 |
| | | | ACSL1 | 0.87476 | 4 | 185676748 | 379 |
| | | | PIK3C2G | 0.88602 | 12 | 18414473 | 1398 |
| | | | PPARGC1A | 0.88709 | 4 | 23793643 | 418 |
| | | | CDKN2A | 0.91225 | 9 | 21967750 | 125 |
| | | | ABCB11 | 0.92614 | 2 | 169779448 | 483 |
| | | | ABCB1 | 0.92683 | 7 | 87133178 | 557 |
| | | | KNG1 | 0.92929 | 3 | 186435097 | 363 |
| | | | C6 | 0.93688 | 5 | 41142247 | 547 |
| | | | SLC27A2 | 0.94331 | 15 | 50474392 | 394 |
| | | | CYP2C8 | 0.95427 | 10 | 96796528 | 293 |
| | | | GSTM4 | 0.95504 | 1 | 110198697 | 138 |
| | | | GADD45A | 0.95666 | 1 | 68150859 | 129 |
| | | | HRG | 0.96356 | 3 | 186383740 | 169 |

| Gene set | Gene set p-value (Hypergeometric test, Bonferroni-corrected) | Number of genes in gene set | Gene name | Gene-based p-value | chr | Start | Number of SNPs |
|---|---|---|---|---|---|---|---|
| | | | FMO4 | 0.96567 | 1 | 171283321 | 136 |
| | | | CYP1A2 | 0.97051 | 15 | 75041183 | 144 |
| | | | SULT2A1 | 0.9764 | 19 | 48373722 | 237 |
| | | | AOX1 | 0.98877 | 2 | 201450730 | 375 |
| | | | GSTM1 | 0.99151 | 1 | 110230417 | 196 |
| | | | NQO2 | 0.99165 | 6 | 3000049 | 408 |
| | | | GADD45B | 0.99243 | 19 | 2476122 | 188 |
| | | | CYP7A1 | 0.99403 | 8 | 59402736 | 127 |
| | | | ACSL5 | 0.99882 | 10 | 114135955 | 206 |
| HSIAO_LIVER_SPECIFIC_GENES | 0.0000199 | 233 | | | | | |
| | | | APOC3 | 7.2E-38 | 11 | 116700623 | 173 |
| | | | APOA1 | 1.23E-29 | 11 | 116706468 | 171 |
| | | | KLKB1 | 5.86E-11 | 4 | 187148671 | 258 |
| | | | F11 | 9.03E-10 | 4 | 187187117 | 222 |
| | | | FMO3 | 0.00234 | 1 | 171060017 | 277 |
| | | | HRSP12 | 0.01276 | 8 | 99114566 | 194 |
| | | | C1S | 0.01508 | 12 | 7167979 | 79 |
| | | | MBL2 | 0.01605 | 10 | 54525139 | 217 |
| | | | ACOX2 | 0.01785 | 3 | 58490862 | 174 |
| | | | CYP2A6 | 0.02038 | 19 | 41349442 | 213 |
| | | | FGFR4 | 0.02077 | 5 | 176516550 | 190 |
| | | | AKR1C1 | 0.03062 | 10 | 5005453 | 300 |
| | | | PCBD1 | 0.04028 | 10 | 72643264 | 163 |
| | | | SAA1 | 0.04225 | 11 | 18287807 | 184 |
| | | | ACAT1 | 0.04427 | 11 | 107992257 | 133 |
| | | | C1R | 0.04702 | 12 | 7187514 | 111 |
| | | | FGG | 0.06555 | 4 | 155525727 | 77 |
| | | | CYP2A7 | 0.06713 | 19 | 41381343 | 280 |
| | | | SAA4 | 0.06727 | 11 | 18252901 | 184 |
| | | | UGT2B4 | 0.07005 | 4 | 70345882 | 505 |
| | | | LCAT | 0.07266 | 16 | 67973786 | 46 |
| | | | CRHBP | 0.09248 | 5 | 76248679 | 201 |

| Gene set | Gene set p-value (Hypergeometric test, Bonferroni-corrected) | Number of genes in gene set | Gene name | Gene-based p-value | chr | Start | Number of SNPs |
|---|---|---|---|---|---|---|---|
| | | | C8B | 0.09652 | 1 | 57394882 | 375 |
| | | | HPN | 0.09852 | 19 | 35531409 | 201 |
| | | | UGT2B7 | 0.09948 | 4 | 69962192 | 278 |
| | | | SERPINC1 | 0.10496 | 1 | 173872941 | 135 |
| | | | APOB | 0.10523 | 2 | 21224300 | 174 |
| | | | BAAT | 0.11249 | 9 | 104122698 | 221 |
| | | | HPD | 0.11632 | 12 | 122277432 | 205 |
| | | | HPX | 0.12314 | 11 | 6452267 | 115 |
| | | | ARG1 | 0.13078 | 6 | 131894343 | 98 |
| | | | PROS1 | 0.13263 | 3 | 93591880 | 148 |
| | | | TST | 0.13698 | 22 | 37406899 | 198 |
| | | | SEPP1 | 0.14069 | 5 | 42799981 | 150 |
| | | | GATM | 0.14447 | 15 | 45653321 | 146 |
| | | | GSTZ1 | 0.14522 | 14 | 77787229 | 155 |
| | | | F13B | 0.14765 | 1 | 197008320 | 157 |
| | | | ACADSB | 0.15142 | 10 | 124768428 | 317 |
| | | | CPS1 | 0.15434 | 2 | 211458078 | 679 |
| | | | VTN | 0.15877 | 17 | 26694298 | 84 |
| | | | CYP4F2 | 0.15966 | 19 | 15988833 | 347 |
| | | | NAMPT | 0.16302 | 7 | 105888731 | 199 |
| | | | NR1H4 | 0.16331 | 12 | 100897137 | 259 |
| | | | F10 | 0.16522 | 13 | 113777112 | 183 |
| | | | MGST2 | 0.17173 | 4 | 140586921 | 394 |
| | | | ALAS1 | 0.17301 | 3 | 52232098 | 72 |
| | | | HSD17B4 | 0.19103 | 5 | 118788191 | 377 |
| | | | AKR1D1 | 0.1957 | 7 | 137761177 | 262 |
| | | | HABP2 | 0.19607 | 10 | 115312777 | 305 |
| | | | ALDOB | 0.19616 | 9 | 104182841 | 211 |
| | | | ORM1 | 0.20744 | 9 | 117085302 | 127 |
| | | | TAT | 0.20781 | 16 | 71600753 | 108 |
| | | | POR | 0.21279 | 7 | 75544419 | 236 |
| | | | LGALS4 | 0.21655 | 19 | 39292310 | 189 |

| Gene set | Gene set p-value (Hypergeometric test, Bonferroni-corrected) | Number of genes in gene set | Gene name | Gene-based p-value | chr | Start | Number of SNPs |
|---|---|---|---|---|---|---|---|
| | | | C4BPB | 0.22218 | 1 | 207262627 | 108 |
| | | | CDH2 | 0.23519 | 18 | 25530926 | 594 |
| | | | CYP2E1 | 0.24962 | 10 | 135340866 | 264 |
| | | | ALDH2 | 0.25057 | 12 | 112204690 | 94 |
| | | | ITIH2 | 0.25357 | 10 | 7745235 | 394 |
| | | | IL13 | 0.25912 | 5 | 131993864 | 81 |
| | | | PCSK6 | 0.26021 | 15 | 101923952 | 1089 |
| | | | GHR | 0.26821 | 5 | 42565965 | 822 |
| | | | PZP | 0.27685 | 12 | 9301435 | 279 |
| | | | CYP27A1 | 0.27923 | 2 | 219646471 | 163 |
| | | | SERPINF2 | 0.28202 | 17 | 1646319 | 211 |
| | | | IGFBP1 | 0.28328 | 7 | 45927958 | 134 |
| | | | SERPINA5 | 0.2877 | 14 | 95047705 | 298 |
| | | | PLOD2 | 0.29236 | 3 | 145787227 | 383 |
| | | | RGL2 | 0.29618 | 6 | 33259430 | 115 |
| | | | SLC10A1 | 0.30116 | 14 | 70242551 | 136 |
| | | | SOD1 | 0.31 | 21 | 33031934 | 124 |
| | | | PON3 | 0.3106 | 7 | 94989183 | 153 |
| | | | SERPINF1 | 0.31498 | 17 | 1665258 | 247 |
| | | | G6PC | 0.32608 | 17 | 41052813 | 76 |
| | | | GPT | 0.32648 | 8 | 145729464 | 73 |
| | | | APOA2 | 0.32657 | 1 | 161192082 | 112 |
| | | | CBS | 0.33032 | 21 | 44473300 | 350 |
| | | | PTGR1 | 0.33412 | 9 | 114325246 | 339 |
| | | | F7 | 0.33884 | 13 | 113760101 | 193 |
| | | | TF | 0.34212 | 3 | 133464976 | 250 |
| | | | F12 | 0.34372 | 5 | 176829138 | 83 |
| | | | CFB | 0.35454 | 6 | 31913720 | 129 |
| | | | GYS2 | 0.36235 | 12 | 21689122 | 394 |
| | | | FMO5 | 0.36946 | 1 | 146657839 | 453 |
| | | | PLGLB2 | 0.37232 | 2 | 88047605 | 14 |
| | | | PYGL | 0.37412 | 14 | 51371934 | 338 |

| Gene set | Gene set p-value (Hypergeometric test, Bonferroni-corrected) | Number of genes in gene set | Gene name | Gene-based p-value | chr | Start | Number of SNPs |
|---|---|---|---|---|---|---|---|
| | | | C4BPA | 0.37606 | 1 | 207277606 | 184 |
| | | | ALDH6A1 | 0.38319 | 14 | 74524367 | 244 |
| | | | KYNU | 0.3906 | 2 | 143635194 | 537 |
| | | | MGST1 | 0.39757 | 12 | 16506350 | 235 |
| | | | HSD11B1 | 0.39862 | 1 | 209878135 | 195 |
| | | | AGXT | 0.39981 | 2 | 241808161 | 280 |
| | | | HMGCL | 0.4065 | 1 | 24128366 | 85 |
| | | | CES2 | 0.41484 | 16 | 66968373 | 77 |
| | | | BDH1 | 0.42601 | 3 | 197236653 | 498 |
| | | | CES1 | 0.42738 | 16 | 55836763 | 294 |
| | | | TAF6 | 0.43814 | 7 | 99704692 | 105 |
| | | | APCS | 0.44131 | 1 | 159557615 | 179 |
| | | | AGTR1 | 0.45479 | 3 | 148447966 | 256 |
| | | | F2 | 0.45845 | 11 | 46740715 | 120 |
| | | | SERPINE1 | 0.46027 | 7 | 100770369 | 188 |
| | | | CYB5A | 0.4699 | 18 | 71920526 | 330 |
| | | | DHCR24 | 0.4701 | 1 | 55315299 | 333 |
| | | | DPYS | 0.47086 | 8 | 105391651 | 385 |
| | | | GSTA2 | 0.47522 | 6 | 52614884 | 187 |
| | | | ABCC6 | 0.47947 | 16 | 16315043 | 340 |
| | | | SLC39A14 | 0.47986 | 8 | 22225049 | 346 |
| | | | FGL1 | 0.48075 | 8 | 17721899 | 509 |
| | | | PLIN2 | 0.48447 | 9 | 19115758 | 190 |
| | | | SUPT4H1 | 0.48841 | 17 | 56422535 | 107 |
| | | | SERPINA1 | 0.4915 | 14 | 94843083 | 260 |
| | | | CYP2J2 | 0.50272 | 1 | 60358979 | 184 |
| | | | SSRP1 | 0.50616 | 11 | 57093458 | 132 |
| | | | ACAA2 | 0.52542 | 18 | 47309873 | 195 |
| | | | APOC2 | 0.52755 | 19 | 45449238 | 151 |
| | | | LIPC | 0.54093 | 15 | 58724174 | 659 |
| | | | SERPIND1 | 0.54159 | 22 | 21128382 | 167 |
| | | | SERPINA3 | 0.54326 | 14 | 95078713 | 253 |

| Gene set | Gene set p-value (Hypergeometric test, Bonferroni-corrected) | Number of genes in gene set | Gene name | Gene-based p-value | chr | Start | Number of SNPs |
|---|---|---|---|---|---|---|---|
| | | | MUT | 0.55365 | 6 | 49398072 | 156 |
| | | | ABCB4 | 0.55526 | 7 | 87031360 | 267 |
| | | | HMGCS2 | 0.56216 | 1 | 120290618 | 148 |
| | | | LPIN2 | 0.56276 | 18 | 2916991 | 448 |
| | | | ACSM3 | 0.5804 | 16 | 20775311 | 144 |
| | | | ANG | 0.5949 | 14 | 21156931 | 219 |
| | | | CYP3A4 | 0.59613 | 7 | 99354582 | 68 |
| | | | RBP4 | 0.60611 | 10 | 95351592 | 207 |
| | | | APOF | 0.61214 | 12 | 56754354 | 38 |
| | | | HGFAC | 0.62211 | 4 | 3443659 | 168 |
| | | | C9 | 0.62631 | 5 | 39284377 | 360 |
| | | | C8A | 0.64583 | 1 | 57320442 | 377 |
| | | | SPP2 | 0.64696 | 2 | 234959345 | 309 |
| | | | CDO1 | 0.65842 | 5 | 115140429 | 120 |
| | | | CFHR2 | 0.65887 | 1 | 196912897 | 129 |
| | | | MSMO1 | 0.66863 | 4 | 166248817 | 207 |
| | | | C2 | 0.68129 | 6 | 31895253 | 254 |
| | | | AKR1C4 | 0.68534 | 10 | 5238797 | 405 |
| | | | ADH1B | 0.68613 | 4 | 100227543 | 179 |
| | | | CP | 0.69549 | 3 | 148890289 | 371 |
| | | | TDO2 | 0.69743 | 4 | 156824844 | 249 |
| | | | RNASE4 | 0.71543 | 14 | 21156935 | 247 |
| | | | AHSG | 0.7164 | 3 | 186330849 | 141 |
| | | | CFHR4 | 0.72714 | 1 | 196857143 | 179 |
| | | | PCK1 | 0.72845 | 20 | 56136136 | 226 |
| | | | CASC3 | 0.73425 | 17 | 38296506 | 126 |
| | | | TTR | 0.73808 | 18 | 29171729 | 103 |
| | | | GSTO1 | 0.74747 | 10 | 106014467 | 109 |
| | | | CAT | 0.75028 | 11 | 34460471 | 283 |
| | | | AZGP1 | 0.75588 | 7 | 99564349 | 135 |
| | | | SLC2A2 | 0.75641 | 3 | 170714136 | 172 |
| | | | APOC4 | 0.76133 | 19 | 45445494 | 147 |

| Gene set | Gene set p-value (Hypergeometric test, Bonferroni-corrected) | Number of genes in gene set | Gene name | Gene-based p-value | chr | Start | Number of SNPs |
|---|---|---|---|---|---|---|---|
| | | | UGT2B15 | 0.77481 | 4 | 69512314 | 287 |
| | | | CYP2D6 | 0.78617 | 22 | 42522500 | 191 |
| | | | ITIH4 | 0.79136 | 3 | 52847005 | 129 |
| | | | ASGR1 | 0.79352 | 17 | 7076750 | 91 |
| | | | ASL | 0.8029 | 7 | 65540833 | 156 |
| | | | SORD | 0.80714 | 15 | 45315301 | 257 |
| | | | ASS1 | 0.80981 | 9 | 133320093 | 357 |
| | | | GGCX | 0.81198 | 2 | 85785847 | 193 |
| | | | NR0B2 | 0.82128 | 1 | 27237974 | 71 |
| | | | LSR | 0.82367 | 19 | 35739558 | 212 |
| | | | NAT2 | 0.82486 | 8 | 18248754 | 333 |
| | | | RARRES2 | 0.82916 | 7 | 150035406 | 135 |
| | | | ITIH3 | 0.82926 | 3 | 52828783 | 116 |
| | | | GLUD1 | 0.83584 | 10 | 88809958 | 209 |
| | | | FST | 0.84512 | 5 | 52776263 | 172 |
| | | | PLG | 0.84724 | 6 | 161123224 | 281 |
| | | | ADH4 | 0.84753 | 4 | 100044807 | 351 |
| | | | EPHX1 | 0.8582 | 1 | 226013001 | 328 |
| | | | AADAC | 0.86311 | 3 | 151531860 | 257 |
| | | | ALB | 0.8635 | 4 | 74269971 | 121 |
| | | | NNMT | 0.86563 | 11 | 114166534 | 150 |
| | | | ITIH1 | 0.86641 | 3 | 52813941 | 112 |
| | | | TFPI | 0.86828 | 2 | 188343304 | 330 |
| | | | CYP2C9 | 0.87177 | 10 | 96698414 | 353 |
| | | | ACSL1 | 0.87476 | 4 | 185676748 | 379 |
| | | | PCCB | 0.8751 | 3 | 135969166 | 208 |
| | | | DDT | 0.88228 | 22 | 24313553 | 213 |
| | | | BZW1 | 0.88698 | 2 | 201677055 | 91 |
| | | | NCOR2 | 0.88962 | 12 | 124808956 | 1036 |
| | | | FABP1 | 0.89514 | 2 | 88422507 | 153 |
| | | | CFI | 0.89635 | 4 | 110661847 | 304 |
| | | | LPA | 0.89897 | 6 | 160952514 | 346 |

| Gene set | Gene set p-value (Hypergeometric test, Bonferroni-corrected) | Number of genes in gene set | Gene name | Gene-based p-value | chr | Start | Number of SNPs |
|---|---|---|---|---|---|---|---|
| | | | APOE | 0.91052 | 19 | 45409657 | 140 |
| | | | HGD | 0.91112 | 3 | 120347014 | 342 |
| | | | GCH1 | 0.91706 | 14 | 55308723 | 317 |
| | | | SLC38A3 | 0.92335 | 3 | 50242678 | 96 |
| | | | FCN2 | 0.92475 | 9 | 137772657 | 242 |
| | | | AFM | 0.92659 | 4 | 74347461 | 178 |
| | | | KNG1 | 0.92929 | 3 | 186435097 | 363 |
| | | | FBP1 | 0.93068 | 9 | 97365420 | 319 |
| | | | HNMT | 0.93144 | 2 | 138721807 | 197 |
| | | | F5 | 0.93674 | 1 | 169481191 | 483 |
| | | | SERPINA6 | 0.9395 | 14 | 94770584 | 207 |
| | | | CRP | 0.94134 | 1 | 159682078 | 99 |
| | | | C5 | 0.94256 | 9 | 123714613 | 334 |
| | | | SEPHS2 | 0.94713 | 16 | 30454945 | 71 |
| | | | BHMT | 0.95279 | 5 | 78407603 | 200 |
| | | | DECR1 | 0.95387 | 8 | 91013579 | 220 |
| | | | PCK2 | 0.95745 | 14 | 24563489 | 79 |
| | | | C8G | 0.95891 | 9 | 139839697 | 155 |
| | | | ABCC2 | 0.95892 | 10 | 101542354 | 277 |
| | | | HRG | 0.96356 | 3 | 186383740 | 169 |
| | | | SC5D | 0.96537 | 11 | 121163576 | 144 |
| | | | HP | 0.96694 | 16 | 72088507 | 132 |
| | | | FAH | 0.96878 | 15 | 80445232 | 239 |
| | | | AGT | 0.96945 | 1 | 230838271 | 261 |
| | | | TM4SF4 | 0.97119 | 3 | 149192367 | 437 |
| | | | SERPING1 | 0.97294 | 11 | 57365704 | 134 |
| | | | SULT2A1 | 0.9764 | 19 | 48373722 | 237 |
| | | | CXCR2 | 0.97662 | 2 | 218990735 | 96 |
| | | | APOH | 0.97663 | 17 | 64208146 | 245 |
| | | | CPB2 | 0.97664 | 13 | 46627321 | 280 |
| | | | RBM4 | 0.97682 | 11 | 66406087 | 76 |
| | | | C3 | 0.98118 | 19 | 6677845 | 342 |

| Gene set | Gene set p-value (Hypergeometric test, Bonferroni-corrected) | Number of genes in gene set | Gene name | Gene-based p-value | chr | Start | Number of SNPs |
|---|---|---|---|---|---|---|---|
| | | | CPN2 | 0.98685 | 3 | 194060493 | 287 |
| | | | ACAA1 | 0.98709 | 3 | 38164200 | 80 |
| | | | ASGR2 | 0.98771 | 17 | 7004640 | 235 |
| | | | AOX1 | 0.98877 | 2 | 201450730 | 375 |
| | | | PROC | 0.98993 | 2 | 128175995 | 150 |
| | | | HAL | 0.9905 | 12 | 96366439 | 272 |
| | | | CYP4A11 | 0.99122 | 1 | 47394845 | 152 |
| | | | ALDH1A1 | 0.99132 | 9 | 75515577 | 198 |
| | | | MTHFD1 | 0.99165 | 14 | 64854758 | 320 |
| | | | ADH6 | 0.99242 | 4 | 100125878 | 155 |
| | | | FGA | 0.99269 | 4 | 155506428 | 78 |
| | | | SHMT1 | 0.99521 | 17 | 18231173 | 213 |
| | | | SDS | 0.99592 | 12 | 113830250 | 112 |
| | | | ATP13A3 | 0.99658 | 3 | 194123402 | 273 |
| | | | FURIN | 0.99704 | 15 | 91416107 | 138 |
| | | | CYP2C18 | 0.99767 | 10 | 96443250 | 237 |
| | | | APOC1 | 0.99865 | 19 | 45417920 | 129 |
| | | | CRADD | 0.999 | 12 | 94071150 | 553 |
| | | | FGB | 0.99946 | 4 | 155484131 | 122 |
| REACTOME_HDL_MEDIATED_LIPID_TRANSPORT | 0.0000506 | 15 | | | | | |
| | | | APOC3 | 7.2E-38 | 11 | 116700623 | 173 |
| | | | APOA1 | 1.23E-29 | 11 | 116706468 | 171 |
| | | | LCAT | 0.07266 | 16 | 67973786 | 46 |
| | | | CETP | 0.13017 | 16 | 56995834 | 235 |
| | | | SCARB1 | 0.14549 | 12 | 125262173 | 458 |
| | | | AMN | 0.21923 | 14 | 103388992 | 135 |
| | | | BMP1 | 0.2825 | 8 | 22022652 | 291 |
| | | | ABCG1 | 0.37514 | 21 | 43640007 | 537 |
| | | | CUBN | 0.46678 | 10 | 16865964 | 1440 |
| | | | APOC2 | 0.52755 | 19 | 45449238 | 151 |
| | | | ABCA1 | 0.72544 | 9 | 107543283 | 775 |
| | | | PLTP | 0.7649 | 20 | 44527258 | 148 |

| Gene set | Gene set p-value (Hypergeometric test, Bonferroni-corrected) | Number of genes in gene set | Gene name | Gene-based p-value | chr | Start | Number of SNPs |
|---|---|---|---|---|---|---|---|
| | | | ALB | 0.8635 | 4 | 74269971 | 121 |
| | | | APOE | 0.91052 | 19 | 45409657 | 140 |
| | | | A2M | 0.96774 | 12 | 9220303 | 194 |
| REACTOME_INTRINSIC_PATHWAY | 0.0000506 | 15 | | | | | |
| | | | KLKB1 | 5.86E-11 | 4 | 187148671 | 258 |
| | | | F11 | 9.03E-10 | 4 | 187187117 | 222 |
| | | | C1QBP | 0.01044 | 17 | 5336098 | 182 |
| | | | GP1BA | 0.13138 | 17 | 4835569 | 115 |
| | | | GP1BB | 0.14987 | 22 | 19711065 | 84 |
| | | | F10 | 0.16522 | 13 | 113777112 | 183 |
| | | | F12 | 0.34372 | 5 | 176829138 | 83 |
| | | | VWF | 0.44368 | 12 | 6058039 | 862 |
| | | | F2 | 0.45845 | 11 | 46740715 | 120 |
| | | | GP9 | 0.63864 | 3 | 128779609 | 49 |
| | | | KNG1 | 0.92929 | 3 | 186435097 | 363 |
| | | | A2M | 0.96774 | 12 | 9220303 | 194 |
| | | | SERPING1 | 0.97294 | 11 | 57365704 | 134 |
| | | | GP5 | 0.98428 | 3 | 194115549 | 194 |
| | | | PRCP | 0.98928 | 11 | 82535408 | 360 |
| MYLLYKANGAS_AMPLIFICATION_HOT_SPOT_23 | 0.0000823 | 19 | | | | | |
| | | | PAFAH1B2 | 1.32E-18 | 11 | 117014999 | 259 |
| | | | PCSK7 | 3.59E-16 | 11 | 117075786 | 225 |
| | | | SDHD | 0.02994 | 11 | 111957547 | 94 |
| | | | ZBTB16 | 0.07566 | 11 | 113931287 | 755 |
| | | | FLI1 | 0.11259 | 11 | 128563810 | 559 |
| | | | PICALM | 0.12433 | 11 | 85668213 | 466 |
| | | | ARHGEF12 | 0.218 | 11 | 120256002 | 359 |
| | | | CBL | 0.22815 | 11 | 119076985 | 194 |
| | | | NUMA1 | 0.3904 | 11 | 71713909 | 250 |
| | | | BIRC3 | 0.56556 | 11 | 102188180 | 116 |
| | | | MAML2 | 0.57217 | 11 | 95709756 | 1392 |
| | | | KMT2A | 0.60235 | 11 | 118307204 | 193 |

| Gene set | Gene set p-value (Hypergeometric test, Bonferroni-corrected) | Number of genes in gene set | Gene name | Gene-based p-value | chr | Start | Number of SNPs |
|---|---|---|---|---|---|---|---|
| | | | CCND1 | 0.66236 | 11 | 69455872 | 138 |
| | | | ATM | 0.84155 | 11 | 108093558 | 288 |
| | | | POU2AF1 | 0.92852 | 11 | 111222980 | 233 |
| | | | CLP1 | 0.93511 | 11 | 57425215 | 69 |
| | | | MEN1 | 0.93969 | 11 | 64570985 | 90 |
| | | | DDX6 | 0.94747 | 11 | 118618472 | 228 |
| | | | DDX10 | 0.99978 | 11 | 108535751 | 612 |
| BIOCARTA_INTRINSIC_PATHWAY | 0.0000823 | 19 | | | | | |
| | | | KLKB1 | 5.86E-11 | 4 | 187148671 | 258 |
| | | | F11 | 9.03E-10 | 4 | 187187117 | 222 |
| | | | F2R | 0.03064 | 5 | 76011867 | 201 |
| | | | FGG | 0.06555 | 4 | 155525727 | 77 |
| | | | COL4A1 | 0.08922 | 13 | 110843087 | 1008 |
| | | | SERPINC1 | 0.10496 | 1 | 173872941 | 135 |
| | | | COL4A2 | 0.12205 | 13 | 110959630 | 1264 |
| | | | PROS1 | 0.13263 | 3 | 93591880 | 148 |
| | | | F10 | 0.16522 | 13 | 113777112 | 183 |
| | | | COL4A4 | 0.18121 | 2 | 227867426 | 645 |
| | | | F12 | 0.34372 | 5 | 176829138 | 83 |
| | | | COL4A3 | 0.35329 | 2 | 228029280 | 972 |
| | | | F2 | 0.45845 | 11 | 46740715 | 120 |
| | | | KNG1 | 0.92929 | 3 | 186435097 | 363 |
| | | | F5 | 0.93674 | 1 | 169481191 | 483 |
| | | | SERPING1 | 0.97294 | 11 | 57365704 | 134 |
| | | | PROC | 0.98993 | 2 | 128175995 | 150 |
| | | | FGA | 0.99269 | 4 | 155506428 | 78 |
| | | | FGB | 0.99946 | 4 | 155484131 | 122 |
| HASLINGER_B_CLL_WITH_11Q23_DELETION | 0.00011 | 22 | | | | | |
| | | | SIK3 | 2.78E-29 | 11 | 116714117 | 1128 |
| | | | PCSK7 | 3.59E-16 | 11 | 117075786 | 225 |
| | | | HS3ST1 | 0.04979 | 4 | 11399987 | 268 |
| | | | CSPG5 | 0.07208 | 3 | 47603727 | 101 |

| Gene set | Gene set p-value (Hypergeometric test, Bonferroni-corrected) | Number of genes in gene set | Gene name | Gene-based p-value | chr | Start | Number of SNPs |
|---|---|---|---|---|---|---|---|
| | | | LPCAT1 | 0.07676 | 5 | 1461541 | 383 |
| | | | PICALM | 0.12433 | 11 | 85668213 | 466 |
| | | | DMXL2 | 0.16869 | 15 | 51739920 | 522 |
| | | | PABPC4 | 0.20457 | 1 | 40026484 | 144 |
| | | | GSN | 0.23708 | 9 | 124062078 | 294 |
| | | | SLC4A7 | 0.24798 | 3 | 27414211 | 515 |
| | | | CTSK | 0.30492 | 1 | 150768683 | 87 |
| | | | FUT8 | 0.47825 | 14 | 65879447 | 938 |
| | | | BIRC2 | 0.47977 | 11 | 102218104 | 162 |
| | | | CNR1 | 0.66202 | 6 | 88849584 | 192 |
| | | | AHR | 0.7134 | 7 | 17338275 | 124 |
| | | | HIST1H2BG | 0.78042 | 6 | 26216427 | 176 |
| | | | UBE4A | 0.78133 | 11 | 118230295 | 137 |
| | | | PPP2R1B | 0.84056 | 11 | 111608604 | 176 |
| | | | ATM | 0.84155 | 11 | 108093558 | 288 |
| | | | CASP1 | 0.90713 | 11 | 104896236 | 181 |
| | | | ADRBK2 | 0.95992 | 22 | 25960860 | 543 |
| | | | DDX10 | 0.99978 | 11 | 108535751 | 612 |
| OHGUCHI_LIVER_HNF4A_TARGETS_DN | 0.00014 | 144 | | | | | |
| | | | APOA4 | 4.06E-38 | 11 | 116691417 | 179 |
| | | | F11 | 9.03E-10 | 4 | 187187117 | 222 |
| | | | CYP4V2 | 1.21E-09 | 4 | 187112673 | 269 |
| | | | SLC22A7 | 0.00013 | 6 | 43265997 | 101 |
| | | | FABP2 | 0.002 | 4 | 120238404 | 274 |
| | | | CEACAM1 | 0.01551 | 19 | 43011457 | 57 |
| | | | MBL2 | 0.01605 | 10 | 54525139 | 217 |
| | | | NECAB1 | 0.01799 | 8 | 91803920 | 444 |
| | | | TMEM30B | 0.02245 | 14 | 61744088 | 112 |
| | | | FAM107B | 0.02539 | 10 | 14560555 | 1243 |
| | | | SARDH | 0.02612 | 9 | 136528683 | 494 |
| | | | ELOVL3 | 0.03382 | 10 | 103986034 | 66 |
| | | | CYP8B1 | 0.04114 | 3 | 42913683 | 123 |

| Gene set | Gene set p-value (Hypergeometric test, Bonferroni-corrected) | Number of genes in gene set | Gene name | Gene-based p-value | chr | Start | Number of SNPs |
|---|---|---|---|---|---|---|---|
| | | | ENPP3 | 0.04155 | 6 | 131958374 | 310 |
| | | | GCGR | 0.06722 | 17 | 79762007 | 72 |
| | | | SAA4 | 0.06727 | 11 | 18252901 | 184 |
| | | | IYD | 0.06921 | 6 | 150690027 | 417 |
| | | | UGT2B4 | 0.07005 | 4 | 70345882 | 505 |
| | | | CCBL1 | 0.09147 | 9 | 131595391 | 217 |
| | | | C8B | 0.09652 | 1 | 57394882 | 375 |
| | | | BAAT | 0.11249 | 9 | 104122698 | 221 |
| | | | KMO | 0.11771 | 1 | 241695433 | 313 |
| | | | SULT1B1 | 0.1393 | 4 | 70592685 | 276 |
| | | | NUDT7 | 0.14281 | 16 | 77756388 | 423 |
| | | | PDZK1 | 0.14564 | 1 | 145727665 | 46 |
| | | | F13B | 0.14765 | 1 | 197008320 | 157 |
| | | | MPDZ | 0.15472 | 9 | 13105702 | 373 |
| | | | PROZ | 0.16373 | 13 | 113812967 | 114 |
| | | | APOM | 0.17091 | 6 | 31623670 | 158 |
| | | | NLRP12 | 0.17415 | 19 | 54296854 | 439 |
| | | | MCM10 | 0.18651 | 10 | 13203553 | 357 |
| | | | PLA2G12B | 0.1895 | 10 | 74694937 | 61 |
| | | | SRD5A1 | 0.19321 | 5 | 6633499 | 314 |
| | | | GJB2 | 0.19452 | 13 | 20761603 | 181 |
| | | | ERBB3 | 0.21321 | 12 | 56473891 | 117 |
| | | | AGMAT | 0.24284 | 1 | 15898193 | 233 |
| | | | HSD3B7 | 0.24654 | 16 | 30996518 | 67 |
| | | | LIFR | 0.24869 | 5 | 38475064 | 275 |
| | | | PCYT2 | 0.26035 | 17 | 79860776 | 29 |
| | | | ARHGAP9 | 0.26285 | 12 | 57866037 | 113 |
| | | | SLC30A10 | 0.26568 | 1 | 220087605 | 150 |
| | | | HSD17B2 | 0.29807 | 16 | 82068857 | 292 |
| | | | EGFR | 0.3089 | 7 | 55086724 | 681 |
| | | | TLCD2 | 0.31206 | 17 | 1606083 | 152 |
| | | | SERPINF1 | 0.31498 | 17 | 1665258 | 247 |

| Gene set | Gene set p-value (Hypergeometric test, Bonferroni-corrected) | Number of genes in gene set | Gene name | Gene-based p-value | chr | Start | Number of SNPs |
|---|---|---|---|---|---|---|---|
| | | | GCHFR | 0.32083 | 15 | 41056284 | 102 |
| | | | INSC | 0.32343 | 11 | 15170647 | 571 |
| | | | F12 | 0.34372 | 5 | 176829138 | 83 |
| | | | AQP8 | 0.35189 | 16 | 25228284 | 182 |
| | | | NR1I3 | 0.36512 | 1 | 161199455 | 153 |
| | | | CFHR1 | 0.36826 | 1 | 196788860 | 282 |
| | | | C4BPA | 0.37606 | 1 | 207277606 | 184 |
| | | | RDH5 | 0.39133 | 12 | 56114150 | 59 |
| | | | HYKK | 0.39367 | 15 | 78799905 | 181 |
| | | | BDH1 | 0.42601 | 3 | 197236653 | 498 |
| | | | B3GALT1 | 0.43352 | 2 | 168675181 | 320 |
| | | | SLC22A9 | 0.44783 | 11 | 63137260 | 275 |
| | | | ABCC6 | 0.47947 | 16 | 16315043 | 340 |
| | | | S100A10 | 0.48769 | 1 | 151955385 | 163 |
| | | | SERPINA1 | 0.4915 | 14 | 94843083 | 260 |
| | | | TKFC | 0.51557 | 11 | 61100653 | 51 |
| | | | SERPINA4 | 0.51603 | 14 | 95027756 | 251 |
| | | | APOC2 | 0.52755 | 19 | 45449238 | 151 |
| | | | HEBP1 | 0.53897 | 12 | 13127798 | 173 |
| | | | LIPC | 0.54093 | 15 | 58724174 | 659 |
| | | | BBOX1 | 0.55166 | 11 | 27062508 | 415 |
| | | | SOAT2 | 0.56002 | 12 | 53497273 | 175 |
| | | | PGLYRP2 | 0.56415 | 19 | 15579456 | 131 |
| | | | ENTPD8 | 0.56736 | 9 | 140328815 | 93 |
| | | | ADRA1B | 0.56932 | 5 | 159343739 | 286 |
| | | | UGT2A3 | 0.56981 | 4 | 69794176 | 255 |
| | | | ABAT | 0.5923 | 16 | 8814572 | 920 |
| | | | SHMT2 | 0.59889 | 12 | 57624148 | 60 |
| | | | FAM35A | 0.6225 | 10 | 88854952 | 322 |
| | | | C9 | 0.62631 | 5 | 39284377 | 360 |
| | | | C8A | 0.64583 | 1 | 57320442 | 377 |
| | | | SPP2 | 0.64696 | 2 | 234959345 | 309 |

| Gene set | Gene set p-value (Hypergeometric test, Bonferroni-corrected) | Number of genes in gene set | Gene name | Gene-based p-value | chr | Start | Number of SNPs |
|---|---|---|---|---|---|---|---|
| | | | ETHE1 | 0.6486 | 19 | 44010870 | 190 |
| | | | RARRES1 | 0.66197 | 3 | 158422439 | 257 |
| | | | CYP7B1 | 0.66661 | 8 | 65508528 | 492 |
| | | | SDR9C7 | 0.66821 | 12 | 57316937 | 135 |
| | | | AADAT | 0.67617 | 4 | 170981372 | 200 |
| | | | SLC23A2 | 0.68744 | 20 | 4833001 | 591 |
| | | | SLC45A3 | 0.7123 | 1 | 205626980 | 230 |
| | | | CA5A | 0.71425 | 16 | 87921624 | 537 |
| | | | NLRP6 | 0.72352 | 11 | 278569 | 211 |
| | | | PNPLA7 | 0.72702 | 9 | 140354403 | 240 |
| | | | TFPI2 | 0.74012 | 7 | 93514708 | 184 |
| | | | MFSD2A | 0.77294 | 1 | 40420783 | 97 |
| | | | TFR2 | 0.77807 | 7 | 100218038 | 100 |
| | | | SLC47A1 | 0.7803 | 17 | 19437166 | 194 |
| | | | AQP11 | 0.79209 | 11 | 77300679 | 154 |
| | | | ASGR1 | 0.79352 | 17 | 7076750 | 91 |
| | | | ZFAND4 | 0.81089 | 10 | 46135153 | 274 |
| | | | CLDN1 | 0.81614 | 3 | 190023489 | 321 |
| | | | MASP1 | 0.81646 | 3 | 186964141 | 380 |
| | | | CIDEB | 0.81663 | 14 | 24774392 | 137 |
| | | | SLC27A5 | 0.81851 | 19 | 59009699 | 114 |
| | | | RARRES2 | 0.82916 | 7 | 150035406 | 135 |
| | | | FAAH | 0.83599 | 1 | 46859938 | 153 |
| | | | SLCO2B1 | 0.84272 | 11 | 74870843 | 273 |
| | | | SPATA2L | 0.84704 | 16 | 89762764 | 185 |
| | | | ADH4 | 0.84753 | 4 | 100044807 | 351 |
| | | | ALDH8A1 | 0.85237 | 6 | 135238527 | 148 |
| | | | CMAHP | 0.85392 | 6 | 25108200 | 424 |
| | | | UGT3A2 | 0.85883 | 5 | 36035118 | 226 |
| | | | ACSM1 | 0.85888 | 16 | 20634558 | 315 |
| | | | DIO1 | 0.86196 | 1 | 54359860 | 205 |
| | | | AADAC | 0.86311 | 3 | 151531860 | 257 |

| Gene set | Gene set p-value (Hypergeometric test, Bonferroni-corrected) | Number of genes in gene set | Gene name | Gene-based p-value | chr | Start | Number of SNPs |
|---|---|---|---|---|---|---|---|
| | | | CYP2F1 | 0.86359 | 19 | 41620352 | 308 |
| | | | PRKD3 | 0.87092 | 2 | 37477645 | 416 |
| | | | PRODH2 | 0.87561 | 19 | 36290891 | 152 |
| | | | GLYAT | 0.87916 | 11 | 58477560 | 254 |
| | | | CFI | 0.89635 | 4 | 110661847 | 304 |
| | | | MBL1P | 0.91641 | 10 | 81679933 | 232 |
| | | | TNFAIP8L1 | 0.92181 | 19 | 4640028 | 241 |
| | | | CTSH | 0.92445 | 15 | 79214091 | 210 |
| | | | CYP2C19 | 0.9256 | 10 | 96522437 | 379 |
| | | | HAO1 | 0.92625 | 20 | 7863630 | 318 |
| | | | PXDC1 | 0.93052 | 6 | 3722835 | 382 |
| | | | TTC39C | 0.93176 | 18 | 21693316 | 477 |
| | | | PERP | 0.93423 | 6 | 138409641 | 225 |
| | | | C6 | 0.93688 | 5 | 41142247 | 547 |
| | | | VWCE | 0.9483 | 11 | 61025757 | 72 |
| | | | TPMT | 0.95143 | 6 | 18128544 | 261 |
| | | | SUCNR1 | 0.95188 | 3 | 151591430 | 150 |
| | | | C8G | 0.95891 | 9 | 139839697 | 155 |
| | | | FITM1 | 0.95902 | 14 | 24600674 | 71 |
| | | | DDC | 0.96031 | 7 | 50560344 | 674 |
| | | | HAAO | 0.9607 | 2 | 42994228 | 256 |
| | | | ASPDH | 0.96246 | 19 | 51014856 | 136 |
| | | | KCNN2 | 0.96285 | 5 | 113769226 | 447 |
| | | | HRG | 0.96356 | 3 | 186383740 | 169 |
| | | | SC5D | 0.96537 | 11 | 121163576 | 144 |
| | | | SERPINA12 | 0.96892 | 14 | 94953619 | 290 |
| | | | ACP5 | 0.97058 | 19 | 11685474 | 145 |
| | | | IL1RAP | 0.9791 | 3 | 190231839 | 618 |
| | | | SLC17A3 | 0.98342 | 6 | 25845327 | 212 |
| | | | CPN2 | 0.98685 | 3 | 194060493 | 287 |
| | | | XYLB | 0.98978 | 3 | 38388250 | 354 |
| | | | QPRT | 0.99476 | 16 | 29690440 | 102 |

| Gene set | Gene set p-value (Hypergeometric test, Bonferroni-corrected) | Number of genes in gene set | Gene name | Gene-based p-value | chr | Start | Number of SNPs |
|---|---|---|---|---|---|---|---|
| | | | SLC3A1 | 0.99557 | 2 | 44502596 | 245 |
| | | | NOX4 | 0.99687 | 11 | 89222020 | 646 |
| | | | TMC7 | 0.99986 | 16 | 18995608 | 403 |
| REACTOME_FORMATION_OF_FIBRIN_CLOT_CLOTTING_CASCADE | 0.00021 | 30 | | | | | |
| | | | KLKB1 | 5.86E-11 | 4 | 187148671 | 258 |
| | | | F11 | 9.03E-10 | 4 | 187187117 | 222 |
| | | | C1QBP | 0.01044 | 17 | 5336098 | 182 |
| | | | FGG | 0.06555 | 4 | 155525727 | 77 |
| | | | PF4 | 0.09802 | 4 | 74846541 | 88 |
| | | | SERPINC1 | 0.10496 | 1 | 173872941 | 135 |
| | | | GP1BA | 0.13138 | 17 | 4835569 | 115 |
| | | | PROS1 | 0.13263 | 3 | 93591880 | 148 |
| | | | F13B | 0.14765 | 1 | 197008320 | 157 |
| | | | GP1BB | 0.14987 | 22 | 19711065 | 84 |
| | | | F10 | 0.16522 | 13 | 113777112 | 183 |
| | | | F7 | 0.33884 | 13 | 113760101 | 193 |
| | | | F12 | 0.34372 | 5 | 176829138 | 83 |
| | | | VWF | 0.44368 | 12 | 6058039 | 862 |
| | | | F2 | 0.45845 | 11 | 46740715 | 120 |
| | | | GP9 | 0.63864 | 3 | 128779609 | 49 |
| | | | F3 | 0.84123 | 1 | 94994731 | 101 |
| | | | TFPI | 0.86828 | 2 | 188343304 | 330 |
| | | | KNG1 | 0.92929 | 3 | 186435097 | 363 |
| | | | F13A1 | 0.93279 | 6 | 6144310 | 879 |
| | | | F5 | 0.93674 | 1 | 169481191 | 483 |
| | | | THBD | 0.95391 | 20 | 23026269 | 122 |
| | | | PF4V1 | 0.9557 | 4 | 74719012 | 141 |
| | | | A2M | 0.96774 | 12 | 9220303 | 194 |
| | | | SERPING1 | 0.97294 | 11 | 57365704 | 134 |
| | | | GP5 | 0.98428 | 3 | 194115549 | 194 |
| | | | PRCP | 0.98928 | 11 | 82535408 | 360 |
| | | | PROC | 0.98993 | 2 | 128175995 | 150 |

| Gene set | Gene set p-value (Hypergeometric test, Bonferroni-corrected) | Number of genes in gene set | Gene name | Gene-based p-value | chr | Start | Number of SNPs |
|---|---|---|---|---|---|---|---|
| | | | FGA | 0.99269 | 4 | 155506428 | 78 |
| | | | FGB | 0.99946 | 4 | 155484131 | 122 |
| LEE_LIVER_CANCER_SURVIVAL_UP | 0.00023 | 171 | | | | | |
| | | | APOA5 | 2.14E-38 | 11 | 116660085 | 134 |
| | | | APOC3 | 7.2E-38 | 11 | 116700623 | 173 |
| | | | KLKB1 | 5.86E-11 | 4 | 187148671 | 258 |
| | | | LEAP2 | 0.0000166 | 5 | 132209357 | 87 |
| | | | FMO3 | 0.00234 | 1 | 171060017 | 277 |
| | | | TTBK1 | 0.00288 | 6 | 43211221 | 230 |
| | | | AMT | 0.00807 | 3 | 49454210 | 98 |
| | | | TMEM176B | 0.01473 | 7 | 150488375 | 211 |
| | | | ACOX2 | 0.01785 | 3 | 58490862 | 174 |
| | | | MSRA | 0.03708 | 8 | 9953061 | 1852 |
| | | | DENND5B | 0.04919 | 12 | 31535156 | 943 |
| | | | CGREF1 | 0.05517 | 2 | 27323461 | 96 |
| | | | KHK | 0.0672 | 2 | 27309610 | 84 |
| | | | UGT2B4 | 0.07005 | 4 | 70345882 | 505 |
| | | | FAM134C | 0.09457 | 17 | 40731525 | 127 |
| | | | LDHAL6A | 0.10043 | 11 | 18477373 | 203 |
| | | | SERPINC1 | 0.10496 | 1 | 173872941 | 135 |
| | | | FLJ30679 | 0.11105 | 16 | 86588925 | 165 |
| | | | MRM1 | 0.11236 | 17 | 34958024 | 124 |
| | | | BAAT | 0.11249 | 9 | 104122698 | 221 |
| | | | HPD | 0.11632 | 12 | 122277432 | 205 |
| | | | SGPL1 | 0.11801 | 10 | 72575703 | 377 |
| | | | MORN1 | 0.12502 | 1 | 2306292 | 446 |
| | | | TTPA | 0.1386 | 8 | 63972047 | 233 |
| | | | STK32B | 0.13916 | 4 | 5053243 | 2322 |
| | | | CABIN1 | 0.14165 | 22 | 24407819 | 366 |
| | | | F13B | 0.14765 | 1 | 197008320 | 157 |
| | | | MPDZ | 0.15472 | 9 | 13105702 | 373 |
| | | | F10 | 0.16522 | 13 | 113777112 | 183 |

| Gene set | Gene set p-value (Hypergeometric test, Bonferroni-corrected) | Number of genes in gene set | Gene name | Gene-based p-value | chr | Start | Number of SNPs |
|---|---|---|---|---|---|---|---|
| | | | ALAS1 | 0.17301 | 3 | 52232098 | 72 |
| | | | HAGH | 0.1825 | 16 | 1859103 | 532 |
| | | | SRD5A1 | 0.19321 | 5 | 6633499 | 314 |
| | | | RUNDC3B | 0.21064 | 7 | 87257728 | 407 |
| | | | NECAB2 | 0.21185 | 16 | 84002236 | 607 |
| | | | C4BPB | 0.22218 | 1 | 207262627 | 108 |
| | | | CUX2 | 0.22606 | 12 | 111471827 | 512 |
| | | | CYP4F11 | 0.23122 | 19 | 16023179 | 321 |
| | | | ACSM5 | 0.25264 | 16 | 20420855 | 346 |
| | | | ZP3 | 0.25956 | 7 | 76054271 | 336 |
| | | | PCSK6 | 0.26021 | 15 | 101923952 | 1089 |
| | | | PCYT2 | 0.26035 | 17 | 79860776 | 29 |
| | | | DEPDC7 | 0.26449 | 11 | 33037727 | 265 |
| | | | CRYL1 | 0.27054 | 13 | 20977805 | 619 |
| | | | ECHS1 | 0.28049 | 10 | 135175986 | 181 |
| | | | FAM169A | 0.28464 | 5 | 74073398 | 289 |
| | | | SEPT4 | 0.28491 | 17 | 56597610 | 112 |
| | | | ABCG5 | 0.28784 | 2 | 44039610 | 296 |
| | | | VPREB1 | 0.30007 | 22 | 22599191 | 267 |
| | | | MASP2 | 0.30869 | 1 | 11104854 | 142 |
| | | | SALL1 | 0.30886 | 16 | 51169885 | 103 |
| | | | INSR | 0.31153 | 19 | 7112265 | 958 |
| | | | SERPINF1 | 0.31498 | 17 | 1665258 | 247 |
| | | | G6PC | 0.32608 | 17 | 41052813 | 76 |
| | | | C1QTNF4 | 0.33232 | 11 | 47611215 | 63 |
| | | | ACSL6 | 0.3356 | 5 | 131285666 | 171 |
| | | | SELENBP1 | 0.33918 | 1 | 151336777 | 94 |
| | | | F12 | 0.34372 | 5 | 176829138 | 83 |
| | | | CPT2 | 0.34885 | 1 | 53662100 | 125 |
| | | | CES3 | 0.35101 | 16 | 67001377 | 97 |
| | | | LPIN1 | 0.35166 | 2 | 11886721 | 672 |
| | | | ANXA9 | 0.35487 | 1 | 150954498 | 139 |

| Gene set | Gene set p-value (Hypergeometric test, Bonferroni-corrected) | Number of genes in gene set | Gene name | Gene-based p-value | chr | Start | Number of SNPs |
|---|---|---|---|---|---|---|---|
| | | | NDUFS2 | 0.35577 | 1 | 161171936 | 123 |
| | | | STARD10 | 0.36599 | 11 | 72465773 | 199 |
| | | | PKLR | 0.36753 | 1 | 155259083 | 80 |
| | | | OGDHL | 0.37142 | 10 | 50942686 | 198 |
| | | | DCXR | 0.37437 | 17 | 79993756 | 126 |
| | | | AMFR | 0.38882 | 16 | 56395363 | 304 |
| | | | RDH5 | 0.39133 | 12 | 56114150 | 59 |
| | | | ABHD6 | 0.39473 | 3 | 58223258 | 386 |
| | | | UNKL | 0.40469 | 16 | 1448305 | 251 |
| | | | CES2 | 0.41484 | 16 | 66968373 | 77 |
| | | | C6orf106 | 0.41775 | 6 | 34555056 | 354 |
| | | | PLPP1 | 0.41981 | 5 | 54720669 | 389 |
| | | | MAP4K1 | 0.42254 | 19 | 39078279 | 154 |
| | | | BDH1 | 0.42601 | 3 | 197236653 | 498 |
| | | | CES1 | 0.42738 | 16 | 55836763 | 294 |
| | | | APCS | 0.44131 | 1 | 159557615 | 179 |
| | | | C1orf115 | 0.44166 | 1 | 220863627 | 229 |
| | | | DPYS | 0.47086 | 8 | 105391651 | 385 |
| | | | MTSS1 | 0.47932 | 8 | 125563010 | 865 |
| | | | SLC35D1 | 0.48225 | 1 | 67465014 | 318 |
| | | | CRABP1 | 0.48296 | 15 | 78632665 | 120 |
| | | | ITPR2 | 0.48378 | 12 | 26488269 | 1882 |
| | | | AMACR | 0.49227 | 5 | 33987090 | 159 |
| | | | CYP2J2 | 0.50272 | 1 | 60358979 | 184 |
| | | | RRN3P1 | 0.50605 | 16 | 21807950 | 62 |
| | | | CAND2 | 0.51572 | 3 | 12838170 | 294 |
| | | | PIPOX | 0.53801 | 17 | 27369917 | 114 |
| | | | HSD17B6 | 0.54112 | 12 | 57157001 | 141 |
| | | | SERPIND1 | 0.54159 | 22 | 21128382 | 167 |
| | | | ABCG8 | 0.56245 | 2 | 44066102 | 447 |
| | | | PINK1 | 0.57519 | 1 | 20959947 | 243 |
| | | | CYP3A4 | 0.59613 | 7 | 99354582 | 68 |

| Gene set | Gene set p-value (Hypergeometric test, Bonferroni-corrected) | Number of genes in gene set | Gene name | Gene-based p-value | chr | Start | Number of SNPs |
|---|---|---|---|---|---|---|---|
| | | | NDRG2 | 0.61144 | 14 | 21484921 | 292 |
| | | | AQP9 | 0.62001 | 15 | 58430407 | 338 |
| | | | ZBTB22 | 0.62054 | 6 | 33282181 | 98 |
| | | | KIRREL2 | 0.63321 | 19 | 36347951 | 122 |
| | | | SLC6A12 | 0.65821 | 12 | 299242 | 280 |
| | | | CDO1 | 0.65842 | 5 | 115140429 | 120 |
| | | | ZBTB20 | 0.66705 | 3 | 114033346 | 1795 |
| | | | ENPP5 | 0.6716 | 6 | 46126918 | 149 |
| | | | C2 | 0.68129 | 6 | 31895253 | 254 |
| | | | ALDH4A1 | 0.68537 | 1 | 19197923 | 367 |
| | | | PRDX6 | 0.69548 | 1 | 173446485 | 112 |
| | | | IVD | 0.72431 | 15 | 40697685 | 145 |
| | | | INSIG1 | 0.72537 | 7 | 155089485 | 279 |
| | | | ABCA6 | 0.72579 | 17 | 67074846 | 210 |
| | | | CRAT | 0.72813 | 9 | 131857072 | 118 |
| | | | PCK1 | 0.72845 | 20 | 56136136 | 226 |
| | | | EGLN2 | 0.72872 | 19 | 41305333 | 185 |
| | | | NF1 | 0.73007 | 17 | 29421944 | 456 |
| | | | SLC2A2 | 0.75641 | 3 | 170714136 | 172 |
| | | | ATG16L1 | 0.76597 | 2 | 234160216 | 237 |
| | | | CFHR5 | 0.76911 | 1 | 196946666 | 151 |
| | | | COBLL1 | 0.77056 | 2 | 165536694 | 630 |
| | | | UGT2B15 | 0.77481 | 4 | 69512314 | 287 |
| | | | MYRIP | 0.77544 | 3 | 40141501 | 1597 |
| | | | CYP2D6 | 0.78617 | 22 | 42522500 | 191 |
| | | | ASGR1 | 0.79352 | 17 | 7076750 | 91 |
| | | | MRPL46 | 0.79377 | 15 | 89002708 | 51 |
| | | | IL6R | 0.7999 | 1 | 154377668 | 258 |
| | | | SLC27A5 | 0.81851 | 19 | 59009699 | 114 |
| | | | SLC22A18 | 0.83881 | 11 | 2923649 | 230 |
| | | | ACADS | 0.84747 | 12 | 121163540 | 199 |
| | | | SEC14L2 | 0.84848 | 22 | 30792929 | 207 |

| Gene set | Gene set p-value (Hypergeometric test, Bonferroni-corrected) | Number of genes in gene set | Gene name | Gene-based p-value | chr | Start | Number of SNPs |
|---|---|---|---|---|---|---|---|
| | | | RHBG | 0.85023 | 1 | 156338979 | 118 |
| | | | AMDHD1 | 0.85382 | 12 | 96337070 | 271 |
| | | | EPHX1 | 0.8582 | 1 | 226013001 | 328 |
| | | | ALDH1L1 | 0.85926 | 3 | 125822403 | 517 |
| | | | DIO1 | 0.86196 | 1 | 54359860 | 205 |
| | | | ITIH1 | 0.86641 | 3 | 52813941 | 112 |
| | | | CLRN1 | 0.86787 | 3 | 150644353 | 276 |
| | | | PAH | 0.86965 | 12 | 103232103 | 433 |
| | | | WNT11 | 0.87245 | 11 | 75897369 | 191 |
| | | | ACSL1 | 0.87476 | 4 | 185676748 | 379 |
| | | | PCCB | 0.8751 | 3 | 135969166 | 208 |
| | | | UGT1A6 | 0.87702 | 2 | 234601511 | 518 |
| | | | SPINK2 | 0.87807 | 4 | 57676025 | 274 |
| | | | GLYAT | 0.87916 | 11 | 58477560 | 254 |
| | | | SLC25A10 | 0.88037 | 17 | 79679265 | 192 |
| | | | HGD | 0.91112 | 3 | 120347014 | 342 |
| | | | TACO1 | 0.9194 | 17 | 61678230 | 41 |
| | | | SLC38A3 | 0.92335 | 3 | 50242678 | 96 |
| | | | HAO1 | 0.92625 | 20 | 7863630 | 318 |
| | | | KNG1 | 0.92929 | 3 | 186435097 | 363 |
| | | | CDHR5 | 0.93781 | 11 | 616564 | 204 |
| | | | ADCK3 | 0.93844 | 1 | 227127937 | 249 |
| | | | TSKU | 0.94036 | 11 | 76494284 | 148 |
| | | | MOGAT1 | 0.94667 | 2 | 223536456 | 248 |
| | | | PC | 0.94819 | 11 | 66615996 | 261 |
| | | | HYAL1 | 0.95348 | 3 | 50337319 | 74 |
| | | | UGT3A1 | 0.95428 | 5 | 35960857 | 234 |
| | | | ABCC2 | 0.95892 | 10 | 101542354 | 277 |
| | | | MST1 | 0.96138 | 3 | 49721379 | 94 |
| | | | AGL | 0.96331 | 1 | 100326765 | 405 |
| | | | MIR1-1HG | 0.9656 | 20 | 61147659 | 322 |
| | | | PARD3B | 0.96683 | 2 | 205410515 | 2893 |

| Gene set | Gene set p-value (Hypergeometric test, Bonferroni-corrected) | Number of genes in gene set | Gene name | Gene-based p-value | chr | Start | Number of SNPs |
|---|---|---|---|---|---|---|---|
| | | | TRIM55 | 0.96904 | 8 | 67039277 | 239 |
| | | | ECHDC2 | 0.97109 | 1 | 53361581 | 93 |
| | | | SERPING1 | 0.97294 | 11 | 57365704 | 134 |
| | | | PHLPP1 | 0.97414 | 18 | 60382671 | 598 |
| | | | SULT2A1 | 0.9764 | 19 | 48373722 | 237 |
| | | | CPB2 | 0.97664 | 13 | 46627321 | 280 |
| | | | CPN2 | 0.98685 | 3 | 194060493 | 287 |
| | | | FBXO31 | 0.98707 | 16 | 87360592 | 369 |
| | | | DSG1 | 0.98836 | 18 | 28898051 | 339 |
| | | | AOX1 | 0.98877 | 2 | 201450730 | 375 |
| | | | MLXIPL | 0.99269 | 7 | 73007523 | 221 |
| | | | EHHADH | 0.99302 | 3 | 184908411 | 229 |
| | | | CCL16 | 0.99599 | 17 | 34303528 | 202 |
| | | | MVK | 0.99811 | 12 | 110011499 | 244 |

**Supplementary Table 6:** HDL-susceptibility SNPs derived from Willer et al. (1) including the original association result on HDL cholesterol and the p-value from the apoA-IV GWA meta-analysis.

| | | | Reported results from original publication | | | | | |
|---|---|---|---|---|---|---|---|---|
| SNP | Chr | position | Reported Gene | n total | effect estimate | effect allele | reported p-value | p-value from apoA-IV GWA[&] |
| rs964184 | 11 | 116648917 | APOA1 | 94000 | 0.106 | C | 6.00E-48 | **0.0001** |
| rs16942887 | 16 | 67928042 | LCAT | 186000 | 0.083 | A | 8.00E-54 | 0.0022 |
| rs4650994 | 1 | 178515312 | ANGPTL1 | 187000 | 0.02 | G | 7.00E-09 | 0.0046 |
| rs7241918 | 18 | 47160953 | LIPG | 93000 | -0.09 | G | 1.00E-44 | 0.0114 |
| rs2290547 | 3 | 47061183 | SETD2 | 187000 | -0.03 | A | 4.00E-09 | 0.0144 |
| rs4142995 | 7 | 17919258 | SNX13 | 165000 | -0.03 | T | 9.00E-12 | 0.0216* |
| rs4765127 | 12 | 124460167 | ZNF664 | 94000 | 0.032 | T | 8.00E-10 | 0.0424 |
| rs11869286 | 17 | 37813856 | STARD3 | 178000 | -0.032 | G | 3.00E-17 | 0.0442* |
| rs4731702 | 7 | 130433384 | KLF14 | 187000 | 0.029 | T | 5.00E-17 | 0.0521 |
| rs4148008 | 17 | 66875294 | ABCA8 | 166000 | -0.028 | G | 1.00E-12 | 0.0594 |
| rs4917014 | 7 | 50305863 | IKZF1 | 187000 | 0.02 | G | 1.00E-08 | 0.0758 |
| rs12678919 | 8 | 19844222 | LPL | 187000 | 0.155 | G | 1.00E-149 | 0.1142* |
| rs7255436 | 19 | 8433196 | ANGPTL4 | 93000 | -0.032 | C | 2.00E-08 | 0.1258 |
| rs838880 | 12 | 125261593 | SCARB1 | 173000 | 0.048 | C | 6.00E-32 | 0.1584 |
| rs13326165 | 3 | 52532118 | STAB1 | 187000 | 0.03 | A | 9.00E-11 | 0.1623 |
| rs4420638 | 19 | 45422946 | APOE | 100000 | -0.067 | G | 2.00E-21 | 0.1780 |
| rs1532085 | 15 | 58683366 | LIPC | 185000 | 0.107 | A | 1.00E-188 | 0.1834 |
| rs2923084 | 11 | 10388782 | AMPD3 | 187000 | -0.026 | G | 5.00E-08 | 0.1907 |
| rs2293889 | 8 | 116599199 | TRPS1 | 180000 | -0.031 | T | 4.00E-17 | 0.1976 |
| rs12967135 | 18 | 57849023 | MC4R | 154000 | -0.026 | A | 4.00E-08 | 0.2071 |
| rs17145738 | 7 | 72982874 | MLXIPL | 185000 | 0.041 | T | 5.00E-13 | 0.2152 |
| rs2925979 | 16 | 81534790 | CMIP | 186000 | -0.035 | T | 1.00E-19 | 0.2277 |
| rs12748152 | 1 | 27138393 | PIGV-NR0B2 | 187000 | -0.05 | T | 1.00E-15 | 0.2281 |
| rs17173637 | 7 | 150529449 | TMEM176A | 184000 | -0.04 | C | 2.00E-08 | 0.2311 |
| rs1121980 | 16 | 53809247 | FTO | 186000 | -0.02 | A | 7.00E-09 | 0.3018* |
| rs605066 | 6 | 139829666 | CITED2 | 94000 | -0.028 | C | 3.00E-08 | 0.3127 |
| rs3764261 | 16 | 56993324 | CETP | 178000 | 0.241 | A | 1.00E-769 | 0.3175 |
| rs2954029 | 8 | 126490972 | TRIB1 | 187000 | 0.04 | T | 3.00E-29 | 0.3220 |
| rs12145743 | 1 | 156700651 | HDGF-PMVK | 181000 | 0.02 | G | 2.00E-08 | 0.3399 |
| rs2013208 | 3 | 50129399 | RBM5 | 170000 | 0.03 | T | 9.00E-12 | 0.3723 |
| rs13076253 | 3 | 131751775 | ACAD11 | 187000 | 0.0283 | A | 5.00E-09 | 0.3749 |
| rs702485 | 7 | 6449272 | DAGLB | 187000 | 0.02 | G | 6.00E-12 | 0.3881* |
| rs1936800 | 6 | 127436064 | RSPO3 | 187000 | 0.02 | C | 3.00E-10 | 0.3956 |
| rs6065906 | 20 | 44554015 | PLTP | 186000 | -0.059 | C | 5.00E-40 | 0.4024 |
| rs12801636 | 11 | 65391317 | KAT5 | 187000 | 0.02 | A | 3.00E-08 | 0.4104 |
| rs4129767 | 17 | 76403984 | PGS1 | 185000 | -0.024 | G | 2.00E-11 | 0.4460* |
| rs174546 | 11 | 61569830 | FADS1-2-3 | 187000 | -0.039 | T | 8.00E-28 | 0.4712 |
| rs2972146 | 2 | 227100698 | IRS1 | 184000 | 0.032 | G | 2.00E-17 | 0.4814 |
| rs7941030 | 11 | 122522375 | UBASH3B | 187000 | 0.027 | C | 1.00E-14 | 0.4970 |
| rs10019888 | 4 | 26062990 | C4orf52 | 187000 | -0.03 | G | 5.00E-08 | 0.5123 |

… to be continued

**Supplementary Table 6:** continued

| | | | Reported results from original publication | | | | | |
|---|---|---|---|---|---|---|---|---|
| SNP | Chr | position | Reported Gene | n total | effect estimate | effect allele | reported p-value | p-value from apoA-IV GWA[&] |
| rs181362 | 22 | 21932068 | UBE2L3 | 178000 | -0.038 | T | 4.00E-18 | 0.5324 |
| rs6805251 | 3 | 119560606 | GSK3B | 186000 | 0.02 | T | 1.00E-08 | 0.5399 |
| rs4846914 | 1 | 230295691 | GALNT2 | 187000 | -0.048 | G | 4.00E-41 | 0.5637 |
| rs11246602 | 11 | 51512090 | OR4C46 | 176000 | 0.03 | C | 2.00E-10 | 0.5641 |
| rs3822072 | 4 | 89741269 | FAM13A | 187000 | -0.03 | A | 4.00E-12 | 0.5749 |
| rs2652834 | 15 | 63396867 | LACTB | 186000 | -0.028 | A | 4.00E-11 | 0.5868 |
| rs998584 | 6 | 43757896 | VEGFA | 184000 | -0.026 | A | 2.00E-11 | 0.5968 |
| rs1689800 | 1 | 182168885 | ZNF648 | 187000 | -0.034 | G | 5.00E-20 | 0.6055 |
| rs3136441 | 11 | 46743247 | LRP4 | 187000 | 0.054 | C | 7.00E-29 | 0.6095 |
| rs7134594 | 12 | 110000193 | MVK | 94000 | -0.035 | C | 2.00E-13 | 0.6132 |
| rs731839 | 19 | 33899065 | PEPD | 185000 | -0.022 | G | 3.00E-09 | 0.6304 |
| rs6450176 | 5 | 53298025 | ARL15 | 187000 | -0.025 | A | 7.00E-10 | 0.6483 |
| rs4759375 | 12 | 123796238 | SBNO1 | 94000 | 0.056 | T | 3.00E-08 | 0.6621 |
| rs1800961 | 20 | 43042364 | HNF4A | 158000 | -0.127 | T | 2.00E-34 | 0.6627 |
| rs4660293 | 1 | 40028180 | PABPC4 | 187000 | -0.035 | G | 3.00E-18 | 0.6827 |
| rs386000 | 19 | 54792761 | LILRA3 | 165000 | 0.048 | C | 3.00E-23 | 0.6950 |
| rs2602836 | 4 | 100014805 | ADH5 | 187000 | 0.02 | A | 5.00E-08 | 0.7217 |
| rs12328675 | 2 | 165540800 | COBLL1 | 187000 | 0.045 | C | 2.00E-15 | 0.7230 |
| rs581080 | 9 | 15305378 | TTC39B | 187000 | -0.042 | G | 1.00E-19 | 0.7231 |
| rs1047891 | 2 | 211540507 | CPS1 | 182000 | -0.03 | A | 9.00E-10 | 0.7985 |
| rs9987289 | 8 | 9183358 | PPP1R3B | 169000 | -0.082 | A | 2.00E-41 | 0.8036 |
| rs1883025 | 9 | 107664301 | ABCA1 | 186000 | -0.07 | T | 2.00E-65 | 0.8095 |
| rs499974 | 11 | 75455021 | MOGAT2-DGAT2 | 187000 | -0.03 | A | 1.00E-08 | 0.8164 |
| rs11613352 | 12 | 57792580 | LRP1 | 187000 | 0.028 | T | 2.00E-13 | 0.8307 |
| rs13107325 | 4 | 103188709 | SLC39A8 | 179000 | -0.071 | T | 1.00E-15 | 0.8452 |
| rs2606736 | 3 | 11400249 | ATG7 | 129000 | 0.03 | C | 5.00E-08 | 0.8523 |
| rs4983559 | 14 | 105277209 | ZBTB42-AKT1 | 184000 | 0.02 | G | 1.00E-08 | 0.8870 |
| rs970548 | 10 | 46013277 | MARCH8-ALOX5 | 187000 | 0.03 | C | 2.00E-10 | 0.9003 |
| rs7134375 | 12 | 20473758 | PDE3A | 187000 | 0.021 | A | 1.00E-08 | 0.9112 |
| rs17695224 | 19 | 52324216 | HAS1 | 185000 | -0.03 | A | 2.00E-13 | 0.9841 |

[&] P-value derived from meta-analysis results of the discovery studies on log-transformed ApoA-IV levels (age-and sex-adjusted); p-values based on a fixed effect( FE) model, besides the one marked with a star (*), where RE model was used. Significant p-values are written in bold font.

**Supplementary Table 7:** TG-susceptibility SNPs, derived from Willer et al. (1) with their original association result on TG and the p-value from the apoA-IV GWA meta-analysis.

| | | | Reported results from original publication (Willer et al.) | | | | | |
|---|---|---|---|---|---|---|---|---|
| SNP | Chr | position | Reported Gene | n total | effect estimate | effect allele | reported p-value | p-value from apoA-IV GWA[&] |
| rs964184 | 11 | 116648917 | APOA1 | 91000 | -0.234 | C | 7.00E-224 | **0.0001** |
| rs10761731 | 10 | 65027610 | JMJD1C | 91000 | -0.031 | T | 8.00E-12 | 0.0039 |
| rs4765127 | 12 | 124460167 | ZNF664 | 91000 | -0.029 | T | 2.00E-08 | 0.0424 |
| rs2068888 | 10 | 94839642 | CYP26A1 | 178000 | -0.024 | A | 2.00E-11 | 0.0555 |
| rs11776767 | 8 | 10683929 | PINX1 | 177000 | 0.022 | C | 3.00E-11 | 0.0958 |
| rs1260326 | 2 | 27730940 | GCKR | 178000 | 0.115 | T | 2.00E-239 | 0.1092 |
| rs12678919 | 8 | 19844222 | LPL | 178000 | -0.17 | G | 2.00E-199 | 0.1142* |
| rs442177 | 4 | 88030261 | KLHL8 | 178000 | -0.031 | G | 1.00E-18 | 0.1230 |
| rs10401969 | 19 | 19407718 | CILP2 | 176000 | -0.121 | C | 1.00E-69 | 0.1351 |
| rs6831256 | 4 | 3473139 | LRPAP1 | 177000 | 0.026 | G | 2.00E-12 | 0.1401 |
| rs1532085 | 15 | 58683366 | LIPC | 176000 | 0.031 | A | 2.00E-18 | 0.1834 |
| rs17145738 | 7 | 72982874 | MLXIPL | 176000 | -0.115 | T | 9.00E-99 | 0.2152 |
| rs2412710 | 15 | 42683787 | CAPN3 | 154000 | 0.099 | A | 2.00E-11 | 0.2219 |
| rs12748152 | 1 | 27138393 | PIGV, NR0B2 | 178000 | 0.037 | T | 1.00E-09 | 0.2281 |
| rs8077889 | 17 | 41878166 | MPP3 | 176000 | 0.025 | C | 1.00E-08 | 0.2333 |
| rs4722551 | 7 | 25991826 | MIR148A | 178000 | 0.023 | C | 9.00E-11 | 0.2603 |
| rs645040 | 3 | 135926622 | MSL2L1 | 178000 | -0.029 | G | 2.00E-12 | 0.2965 |
| rs1121980 | 16 | 53809247 | FTO | 155000 | 0.021 | A | 3.00E-08 | 0.3018* |
| rs3764261 | 16 | 56993324 | CETP | 169000 | -0.04 | A | 2.00E-25 | 0.3175 |
| rs2954029 | 8 | 126490972 | TRIB1 | 178000 | -0.076 | T | 1.00E-107 | 0.3220 |
| rs1936800 | 6 | 127436064 | RSPO3 | 168000 | -0.02 | C | 3.00E-08 | 0.3956 |
| rs6065906 | 20 | 44554015 | PLTP | 176000 | 0.053 | C | 2.00E-34 | 0.4024 |
| rs174546 | 11 | 61569830 | FADS1, FADS2, FADS3 | 178000 | 0.045 | T | 7.00E-38 | 0.4712 |
| rs2972146 | 2 | 227100698 | IRS1 | 175000 | -0.028 | G | 3.00E-15 | 0.4814 |
| rs7248104 | 19 | 7224431 | INSR | 176000 | -0.022 | A | 5.00E-10 | 0.4870 |
| rs9686661 | 5 | 55861786 | MAP3K1 | 177000 | 0.038 | T | 3.00E-16 | 0.4889 |
| rs2131925 | 1 | 63025942 | ANGPTL3 | 178000 | -0.066 | G | 3.00E-74 | 0.4977 |
| rs38855 | 7 | 116358044 | MET | 178000 | -0.019 | G | 2.00E-08 | 0.5377 |
| rs4846914 | 1 | 230295691 | GALNT2 | 178000 | 0.04 | G | 7.00E-31 | 0.5637 |
| rs998584 | 6 | 43757896 | VEGFA | 175000 | 0.029 | A | 3.00E-15 | 0.5968 |
| rs731839 | 19 | 33899065 | PEPD | 176000 | 0.022 | G | 3.00E-09 | 0.6304 |
| rs2929282 | 15 | 44245931 | FRMD5 | 84000 | 0.072 | T | 2.00E-09 | 0.6703 |
| rs3198697 | 16 | 15129940 | PDXDC1 | 176000 | -0.02 | T | 2.00E-08 | 0.6871 |
| rs5756931 | 22 | 38546033 | PLA2G6 | 174000 | -0.02 | C | 3.00E-08 | 0.7652 |
| rs6882076 | 5 | 156390297 | TIMD4 | 178000 | -0.029 | T | 2.00E-15 | 0.8102 |
| rs11613352 | 12 | 57792580 | LRP1 | 178000 | -0.028 | T | 9.00E-14 | 0.8307 |
| rs1832007 | 10 | 5254847 | AKR1C4 | 178000 | -0.033 | G | 2.00E-12 | 0.9346 |
| rs1495741 | 8 | 18272881 | NAT2 | 88000 | 0.04 | G | 3.00E-12 | 0.9535 |

[&] P-value derived from meta-analysis results of the discovery studies on log-transformed ApoA-IV levels (age-and sex-adjusted); p-values based on a fixed effect( FE) model, besides the one marked with a star (*), where RE model was used. Significant p-values are written in bold font.

**Supplementary Table 8:** Kidney function-susceptibility SNPs, derived from Pattaro et al. (2) with their original association result on eGFRcrea and the p-value from the apoA-IV GWA meta-analysis.

| | | | Reported results from original publication (Willer et al.) | | | | | |
|---|---|---|---|---|---|---|---|---|
| SNP | Chr | position | Reported Gene | n total | effect estimate | effect allele | reported p-value | p-value from apoA-IV GWA |
| rs13329952 | 16 | 20366507 | UMOD | 133413 | -0.0158 | T | 9.47E-43 | 0.0069 |
| rs807601 | 2 | 15793014 | DDX1 | 133413 | 0.0064 | T | 6.60E-12 | 0.0115 |
| rs716877 | 13 | 72347448 | DACH1 | 133413 | 0.0049 | C | 6.22E-08 | 0.0317 |
| rs12460876 | 19 | 33356891 | SLC7A9 | 133413 | -0.0066 | T | 1.86E-13 | 0.0414 |
| rs11657044 | 17 | 59450105 | BCAS3 | 133413 | -0.0115 | T | 7.89E-22 | 0.0486 |
| rs17216707 | 20 | 52732362 | BCAS1 | 175579 | -0.0077 | T | 8.83E-15 | 0.0533 |
| rs9916302 | 17 | 37499949 | CDK12 / FBXL20 | 133413 | -0.008 | T | 4.78E-15 | 0.0631 |
| rs2467853 | 15 | 45698793 | GATM | 133413 | 0.0126 | T | 1.05E-42 | 0.0636 |
| rs4014195 | 11 | 65506822 | AP5B1 | 175579 | 0.0055 | C | 1.10E-11 | 0.0970 |
| rs1260326 | 2 | 27730940 | GCKR | 133413 | 0.0068 | T | 3.38E-14 | 0.1092 |
| rs3758086 | 8 | 23714992 | STC1 | 133413 | -0.0071 | A | 1.71E-15 | 0.1161 |
| rs2861422 | 3 | 141724644 | TFDP2 | 133413 | 0.0074 | T | 9.12E-14 | 0.1226 |
| rs17319721 | 4 | 77368847 | SHROOM3 | 133413 | -0.0114 | A | 1.32E-37 | 0.1266 |
| rs10277115 | 7 | 1285195 | UNCX | 175579 | 0.009 | A | 8.72E-14 | 0.1315 |
| rs267734 | 1 | 150951477 | LASS2 | 133413 | -0.0079 | T | 4.01E-13 | 0.1740 |
| rs6420094 | 5 | 176817636 | SLC34A1 | 133413 | 0.0096 | A | 4.92E-22 | 0.1754 |
| rs2802729 | 1 | 243501763 | SDCCAG8 | 175579 | -0.0046 | A | 2.20E-08 | 0.1819 |
| rs4744712 | 9 | 71434707 | PIP5K1B | 133413 | -0.0071 | A | 4.29E-15 | 0.1918 |
| rs4667594 | 2 | 170008506 | LRP2 | 175579 | -0.0044 | A | 3.52E-08 | 0.2112 |
| rs228611 | 4 | 103561709 | NFKB1 | 175579 | -0.0056 | A | 3.58E-12 | 0.2149 |
| rs10491967 | 12 | 3368093 | TSPAN9 | 175579 | -0.0095 | A | 5.18E-14 | 0.2285 |
| rs3750082 | 7 | 32919927 | KBTBD2 | 175579 | 0.0045 | A | 3.22E-08 | 0.2509 |
| rs163160 | 11 | 2789955 | KCNQ1 | 154881 | 0.0065 | A | 2.26E-09 | 0.2710 |
| rs164748 | 16 | 89708292 | DPEP1 | 154881 | 0.0046 | C | 1.95E-08 | 0.2925 |
| rs8091180 | 18 | 77164243 | NFATC1 | 154881 | -0.006 | A | 1.28E-09 | 0.2970 |
| rs848490 | 7 | 77555005 | TMEM60 | 133413 | 0.0073 | C | 7.80E-13 | 0.3122 |
| rs3850625 | 1 | 201016296 | CACNA1S | 154881 | 0.0083 | A | 6.82E-11 | 0.3264 |
| rs12136063 | 1 | 110014170 | SYPL2 | 175579 | 0.0045 | A | 4.71E-08 | 0.3633 |
| rs963837 | 11 | 30749090 | MPPED2 | 133413 | -0.0078 | T | 5.69E-18 | 0.3795 |
| rs1800615 | 1 | 15832281 | CASP9 | 133413 | -0.0058 | T | 1.90E-09 | 0.4011 |
| rs6088580 | 20 | 33285053 | TP53INP2 | 175579 | -0.0049 | C | 1.79E-09 | 0.4764 |
| rs9682041 | 3 | 170091902 | SKIL | 154881 | -0.0068 | T | 2.58E-08 | 0.4933 |
| rs7805747 | 7 | 151407801 | PRKAG2 | 133413 | -0.013 | A | 7.96E-29 | 0.4942 |
| rs7956634 | 12 | 15321194 | PTPRO | 175579 | -0.0068 | T | 7.17E-12 | 0.5277 |
| rs6795744 | 3 | 13906850 | WNT7A | 175579 | 0.006 | A | 3.33E-08 | 0.5444 |
| rs6459680 | 7 | 156258568 | RNF32 | 175579 | -0.0055 | T | 1.07E-09 | 0.5552 |
| rs9472135 | 6 | 43809802 | VEGFA | 133413 | -0.008 | T | 3.34E-15 | 0.5823 |
| rs7422339 / rs1047891 | 2 | 211540507 | CPS1 | 133413 | -0.0106 | A | 2.18E-23 | 0.7985 |
| rs316009 | 6 | 160675764 | SLC22A2 | 133413 | 0.0131 | T | 4.38E-19 | 0.8113 |
| rs1106766 | 12 | 57809456 | INHBC | 175579 | 0.0061 | T | 2.41E-09 | 0.8303 |

… to be continued

**Supplementary Table 8:** continued

| | | | Reported results from original publication (Willer et al.) | | | | | |
|---|---|---|---|---|---|---|---|---|
| SNP | Chr | position | Reported Gene | n total | effect estimate | effect allele | reported p-value | p-value from apoA-IV GWA |
| rs11959928 | 5 | 39397132 | DAB2 | 133413 | -0.0083 | A | 1.66E-20 | 0.8314 |
| rs11666497 | 19 | 38464262 | SIPA1L3 | 175579 | -0.0058 | T | 4.25E-08 | 0.8406 |
| rs491567 | 15 | 53946593 | WDR72 | 133413 | -0.0084 | A | 2.86E-15 | 0.8697 |
| rs1394125 | 15 | 76158983 | UBE2Q2 | 133413 | -0.0073 | A | 5.47E-14 | 0.9061 |
| rs476633 | 15 | 41392134 | INO80 | 133413 | 0.0051 | C | 8.90E-09 | 0.9220 |
| rs6546838 | 2 | 73679280 | ALMS1 | 133413 | -0.0093 | A | 7.72E-20 | 0.9222 |
| rs1044261 | 10 | 1065710 | WDR37 | 133413 | -0.0113 | T | 1.21E-11 | 0.9339 |
| rs2453580 | 17 | 19438321 | SLC47A1 | 133413 | 0.0064 | T | 2.93E-11 | 0.9433 |
| rs10774021 | 12 | 349298 | SLC6A13 | 133413 | -0.0063 | T | 4.77E-12 | 0.9478 |
| rs2712184 | 2 | 217682779 | IGFBP5 | 154881 | -0.0053 | A | 1.33E-10 | 0.9498 |
| rs10994860 | 10 | 52645424 | A1CF | 154881 | 0.0077 | T | 1.07E-12 | 0.9694 |
| rs10513801 | 3 | 185822353 | ETV5 | 154881 | 0.0072 | T | 1.03E-09 | 0.9805 |
| rs7759001 | 6 | 27341409 | ZNF204 | 175579 | -0.0051 | A | 1.75E-08 | 0.9842 |

[&]P-value derived from meta-analysis results of the discovery studies on log-transformed ApoA-IV levels (age-and sex-adjusted); p-values based on a fixed effect( FE) model, besides the one marked with a star (*), where RE model was used. Significant p-values are written in bold font.

## Supplementary Figures

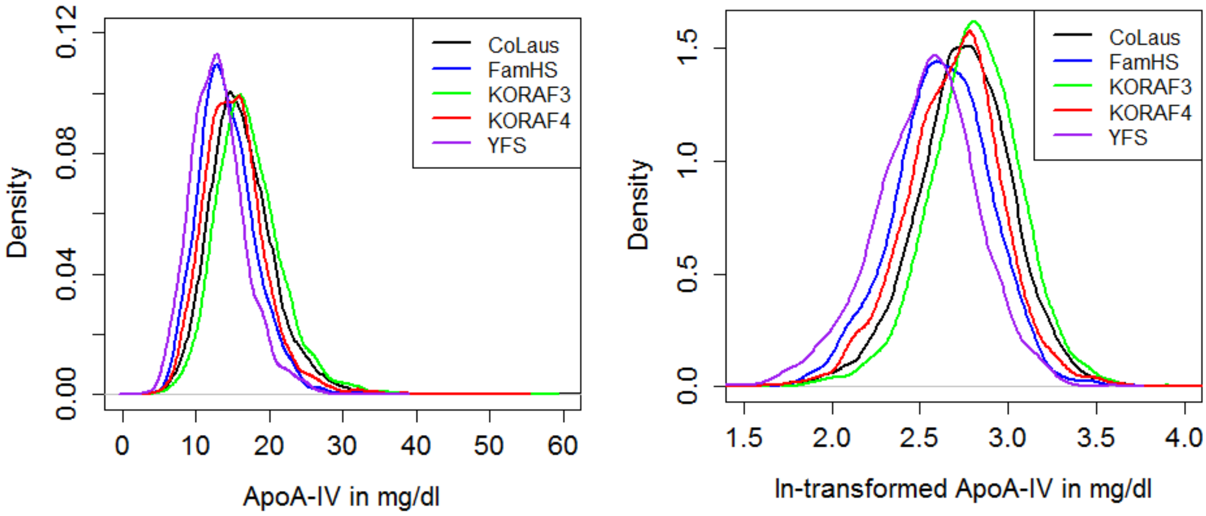


**Supplementary Figure 1:** Distribution of apoA-IV concentrations in all discovery cohorts

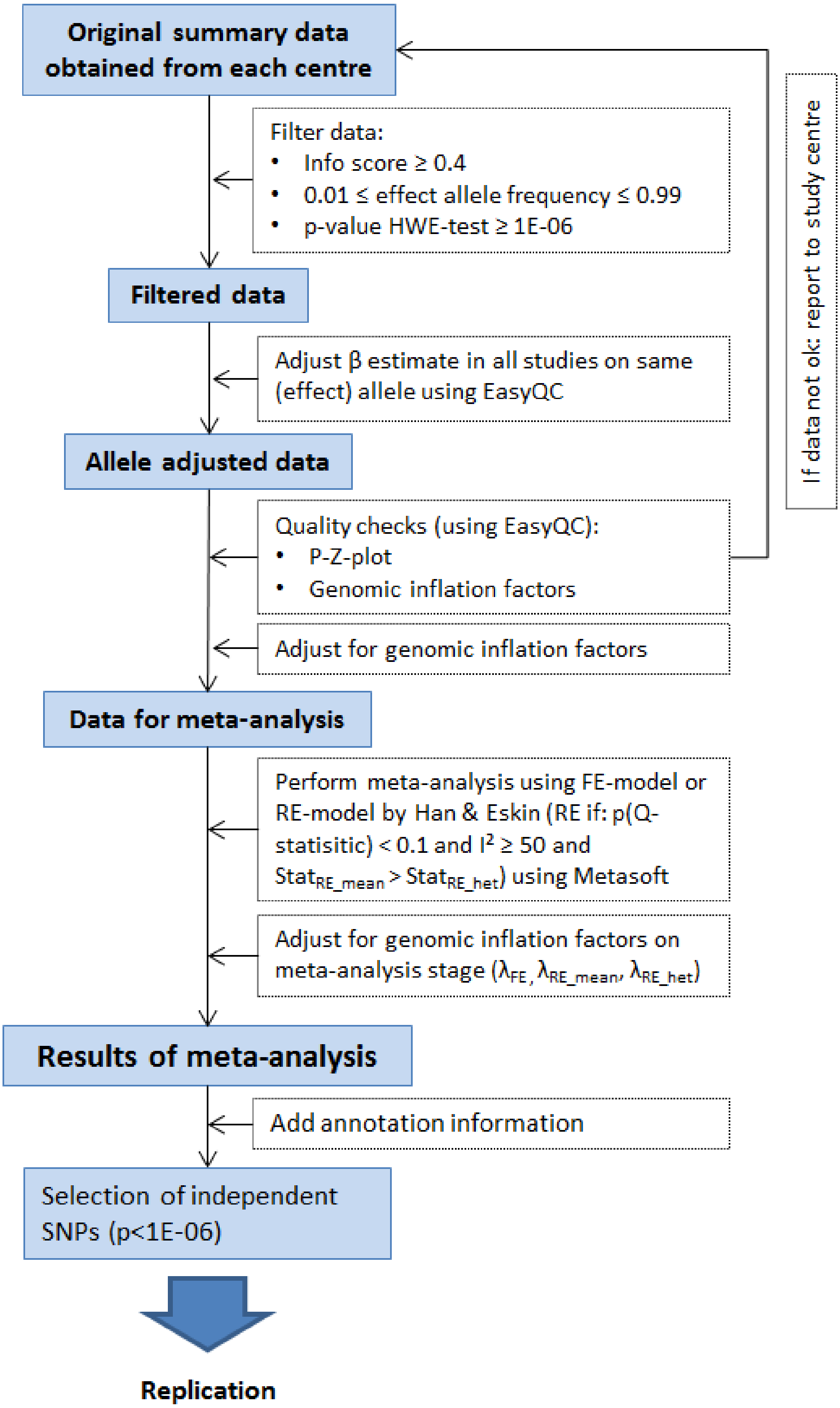


**Supplementary Figure 2:** Quality control and Meta-Analysis Workflow

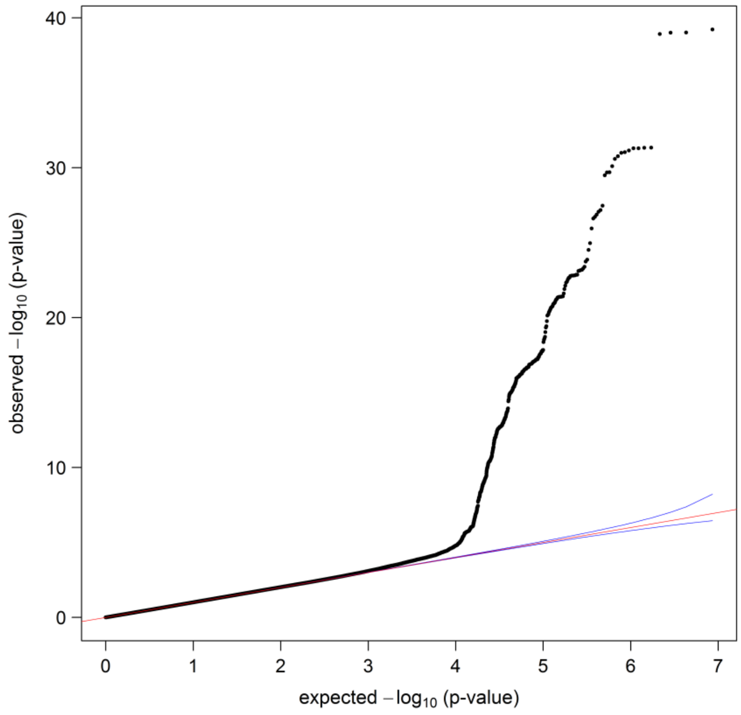


**Supplementary Figure 3:** QQ-plot for the meta-analysis on log-transformed apoA-IV values

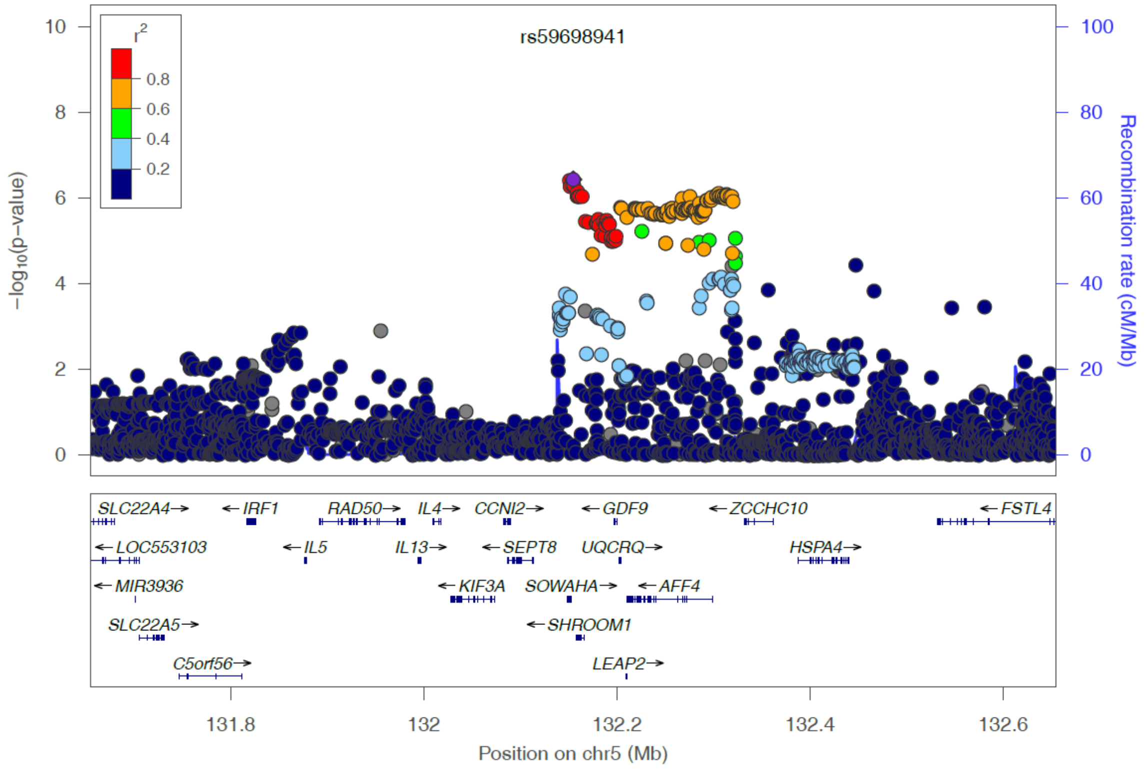


**Supplementary Figure 4:** Regional plot showing the genomic region defined by the *SOWAHA*-lead SNP rs59698941 +/- 500 kB (LD refers to rs59698941, based on 1000G EUR); p-values are derived from the meta-analysis on the five discovery cohorts on log-transformed apoA-IV concentrations.

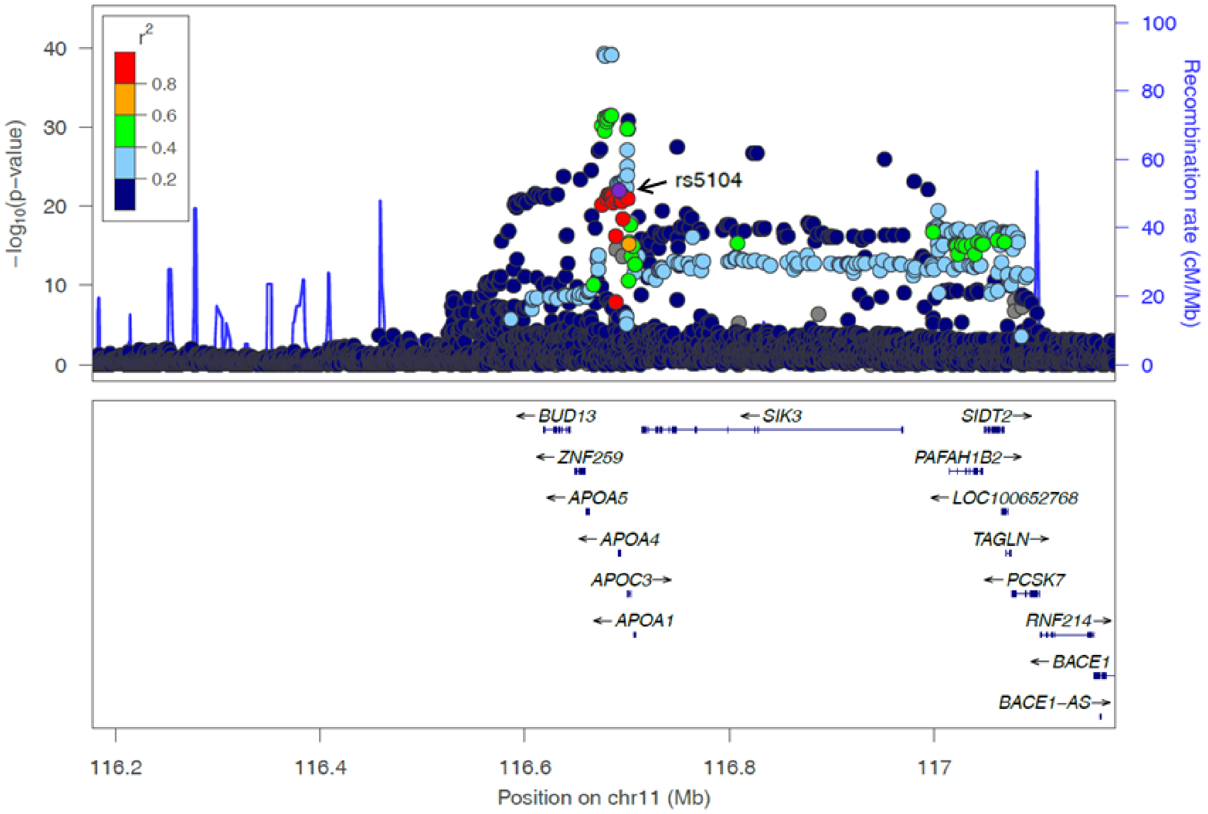


**Supplementary Figure 5:** Regional plot showing the genomic region defined by the *APOA4*-lead SNP rs1729407 +/- 500 kB (LD refers to rs5104, based on 1000G EUR); p-values are derived from the meta-analysis on the five discovery cohorts on log-transformed apoA-IV concentrations.

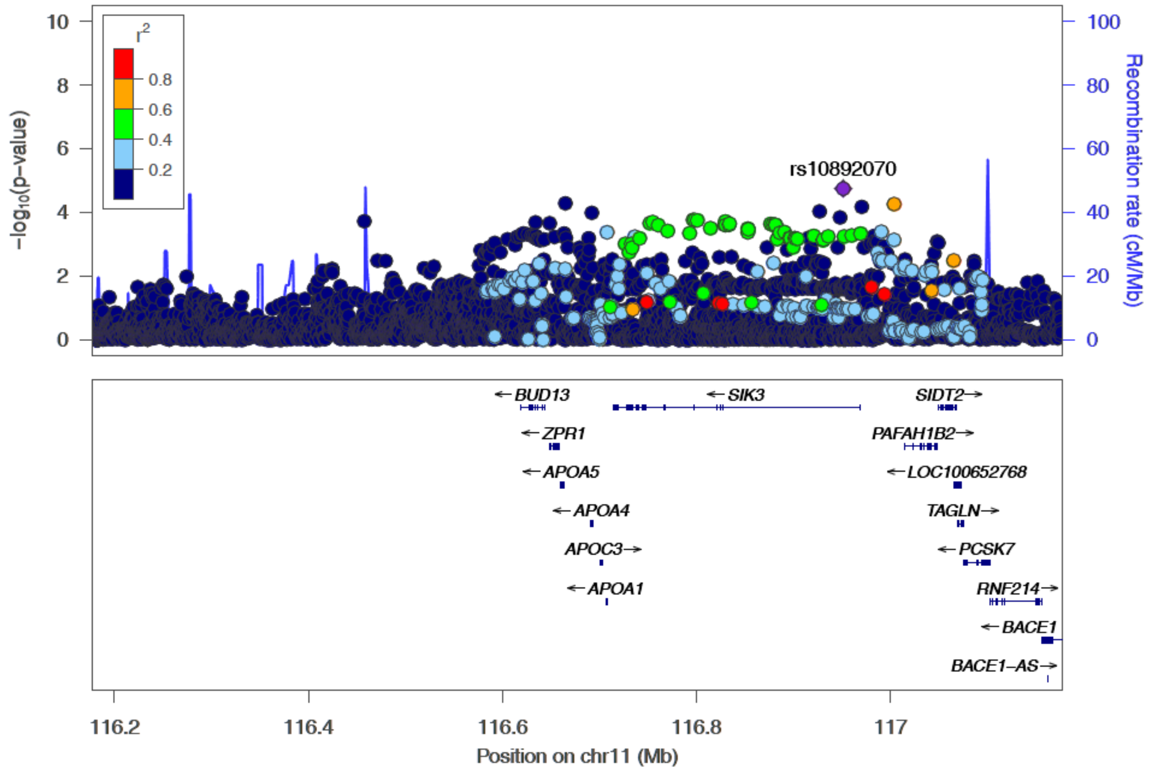


**Supplementary Figure 6:** Regional plot showing the genomic region defined by the *APOA4*-lead SNP rs1729407 +/- 500 kB (LD refers to rs10892070, based on 1000G EUR); p-values are derived from a conditional analysis, adjusting for rs1729407 and rs5104 (based on meta-analyzed results from the five discovery cohorts).

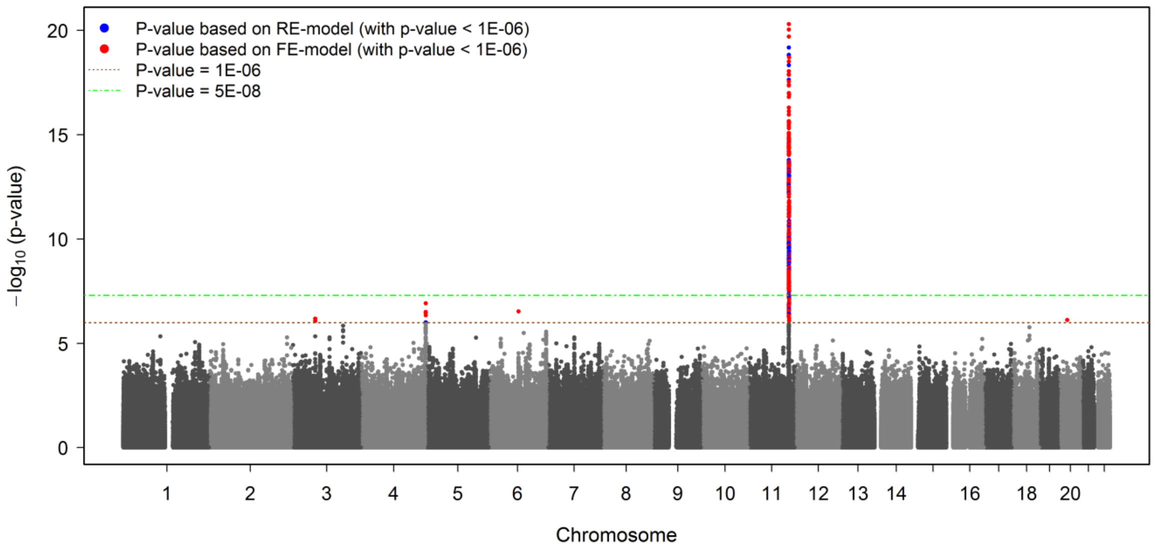


**Supplementary Figure 7:** Manhattan-plot for the meta-analysis on log-transformed apoA-IV values, only in men, based on five discovery cohorts.

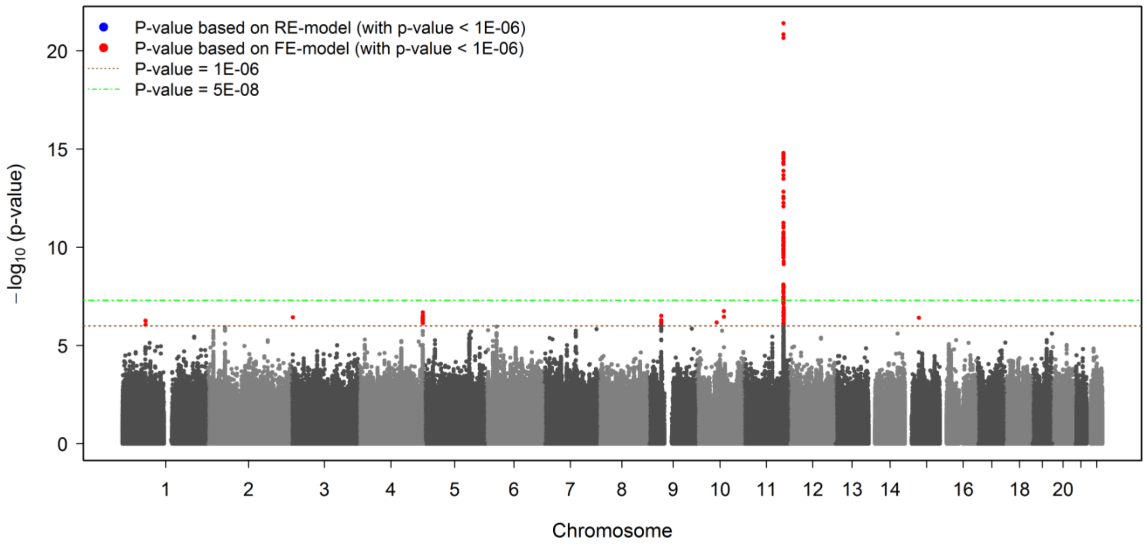


**Supplementary Figure 8:** Manhattan-plot for the meta-analysis on log-transformed apoA-IV values, only in women, based on five discovery cohorts.

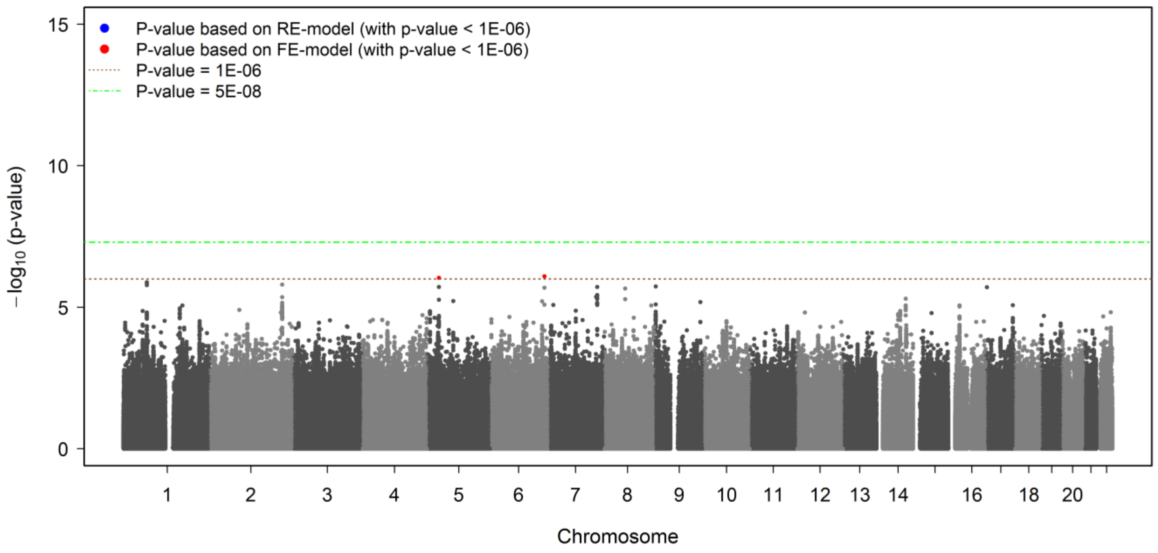


**Supplementary Figure 9:** Manhattan-plot for the meta-analysis on gender-interaction effects on log-transformed apoA-IV values, based on the five discovery cohorts.

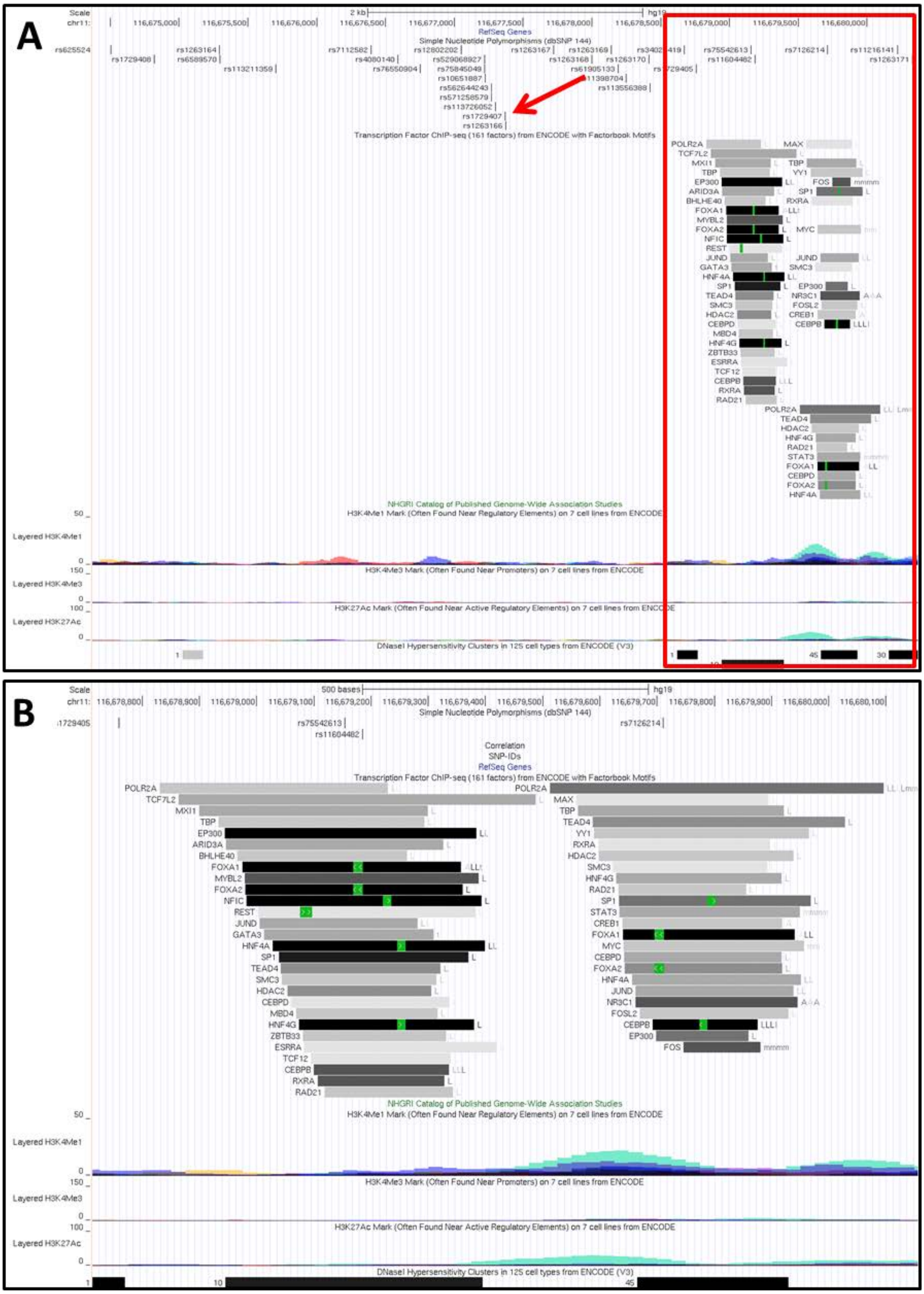


**Supplementary Figure 10:** Regulatory chromatin elements from ENCODE near rs1729407. Panel A: Six kb large region centered on rs1729407 (indicated by a red arrow.)The top row shows the location of all SNPs from dbSNP144 with a MAF > 1%. The rows below indicate the binding sites of transcription factors (grey to black boxes), known GWAS hits (green, no hits present in the figure), regulatory element-specific histone marks, promoter-specific histone marks, active regulatory elements-specific histone marks and DNAse I hypersensitivity sites. Data was retrieved from the UCSC Genome browser (3) hg19 as described before (4). A large cluster of transcription factor

binding sites is present about 1-1.5 kb downstream of rs1729407 (red box) Panel B. Enlarged transcription factor binding sites cluster. Four SNPs with a MAF > 1% are located in this region. The $r^2$ (D') with rs1729407 are: rs1729405: $r^2$=1(1); rs75542613: $r^2$<0.1 (n.a.) rs11604482: no data in SNiPA; rs7216214: $r^2$=0.1 (-1).

## Supplementary References


1. Willer,C.J., Schmidt,E.M., Sengupta,S., Peloso,G.M., Gustafsson,S., Kanoni,S., Ganna,A., Chen,J., Buchkovich,M.L., Mora,S., *et al.* (2013) Discovery and refinement of loci associated with lipid levels. *Nat. Genet.*, **45**, 1274–83.

2. Pattaro,C., Teumer,A., Gorski,M., Chu,A.Y., Li,M., Mijatovic,V., Garnaas,M., Tin,A., Sorice,R., Li,Y., *et al.* (2016) Genetic associations at 53 loci highlight cell types and biological pathways relevant for kidney function. *Nat. Commun.*, **7**.

3. Rosenbloom,K.R., Dreszer,T.R., Pheasant,M., Barber,G.P., Meyer,L.R., Pohl,A., Raney,B.J., Wang,T., Hinrichs,A.S., Zweig,A.S., *et al.* (2010) ENCODE whole-genome data in the UCSC Genome Browser. *Nucleic Acids Res.*, **38**, D620–5.

4. Lamina,C., Coassin,S., Illig,T. and Kronenberg,F. (2011) Look beyond one's own nose: combination of information from publicly available sources reveals an association of GATA4 polymorphisms with plasma triglycerides. *Atherosclerosis*, **219**, 698–703.
